# Semantic Deep Learning: Prior Knowledge and a Type of Four-Term Embedding Analogy to Acquire Treatments for Well-Known Diseases

**DOI:** 10.2196/16948

**Published:** 2020-08-06

**Authors:** Mercedes Arguello Casteleiro, Julio Des Diz, Nava Maroto, Maria Jesus Fernandez Prieto, Simon Peters, Chris Wroe, Carlos Sevillano Torrado, Diego Maseda Fernandez, Robert Stevens

**Affiliations:** 1 Department of Computer Science University of Manchester Manchester United Kingdom; 2 Hospital do Salnés Villagarcía de Arousa Spain; 3 Departamento de Lingüística Aplicada a la Ciencia y a la Tecnología Universidad Politécnica de Madrid Madrid Spain; 4 Salford Languages University of Salford Salford United Kingdom; 5 School of Social Sciences University of Manchester Manchester United Kingdom; 6 BMJ London United Kingdom; 7 Mid Cheshire Hospital Foundation Trust NHS England Crewe United Kingdom

**Keywords:** evidence-based practice, artificial intelligence, deep learning, semantic deep learning, analogical reasoning, embedding analogies, PubMed

## Abstract

**Background:**

How to treat a disease remains to be the most common type of clinical question. Obtaining evidence-based answers from biomedical literature is difficult. Analogical reasoning with embeddings from deep learning (embedding analogies) may extract such biomedical facts, although the state-of-the-art focuses on pair-based proportional (pairwise) analogies such as man:woman::king:queen (“*queen = −man +king +woman*”).

**Objective:**

This study aimed to systematically extract disease treatment statements with a Semantic Deep Learning (SemDeep) approach underpinned by prior knowledge and another type of 4-term analogy (other than pairwise).

**Methods:**

As preliminaries, we investigated Continuous Bag-of-Words (CBOW) embedding analogies in a common-English corpus with five lines of text and observed a type of 4-term analogy (not pairwise) applying the 3CosAdd formula and relating the semantic fields *person* and *death*: “dagger = −Romeo +die +died” (search query: −*Romeo +die +died*). Our SemDeep approach worked with pre-existing items of knowledge (what is known) to make inferences sanctioned by a 4-term analogy (search query −*x +z1 +z2*) from CBOW and Skip-gram embeddings created with a PubMed systematic reviews subset (PMSB dataset). Stage1: Knowledge acquisition. Obtaining a set of terms, candidate y, from embeddings using vector arithmetic. Some n-gram pairs from the cosine and validated with evidence (prior knowledge) are the input for the 3cosAdd, seeking a type of 4-term analogy relating the semantic fields disease and treatment. Stage 2: Knowledge organization. Identification of candidates sanctioned by the analogy belonging to the semantic field treatment and mapping these candidates to unified medical language system Metathesaurus concepts with MetaMap. A concept pair is a brief disease treatment statement (biomedical fact). Stage 3: Knowledge validation. An evidence-based evaluation followed by human validation of biomedical facts potentially useful for clinicians.

**Results:**

We obtained 5352 n-gram pairs from 446 search queries by applying the 3CosAdd. The microaveraging performance of MetaMap for candidate *y* belonging to the semantic field *treatment* was F-measure=80.00% (precision=77.00%, recall=83.25%). We developed an empirical heuristic with some predictive power for *clinical winners*, that is, search queries bringing candidate *y* with evidence of a therapeutic intent for target disease *x*. The search queries *-asthma +inhaled_corticosteroids +inhaled_corticosteroid* and *-epilepsy +valproate +antiepileptic_drug* were *clinical winners*, finding eight evidence-based beneficial treatments.

**Conclusions:**

Extracting treatments with therapeutic intent by analogical reasoning from embeddings (423K n-grams from the PMSB dataset) is an ambitious goal. Our SemDeep approach is knowledge-based, underpinned by embedding analogies that exploit prior knowledge. Biomedical facts from embedding analogies (4-term type, not pairwise) are potentially useful for clinicians. The heuristic offers a practical way to discover beneficial treatments for well-known diseases. Learning from deep learning models does not require a massive amount of data. Embedding analogies are not limited to pairwise analogies; hence, analogical reasoning with embeddings is underexploited.

## Introduction

How to treat a disease or condition remains to be the most common type of clinical question [1]. It is difficult for clinicians to obtain comprehensive information on the clinical (and economic) worth of alternative drug choices for a given condition [2]. Evidence-based biomedical literature, although available in electronic form, primarily remains to be expert-to-expert communication—natural language statements intended for human consumption.

Analogical reasoning is basic relational reasoning without explicit representations of relations [3]. An acknowledged semantic property of embeddings (ie, vectors representing terms) from deep learning [4] is “*their ability to capture relational meanings*” [5], the so-called analogies [6]. Current efforts in analogical reasoning with embeddings focus on pair-based proportional analogies [5,7,8]. This is a type of “*the four-term analogy*” [6], also known as the cross-mapping analogy [6]. An example is *queen = −man +king +woman* [9], also represented as man:woman::king:queen [10], and read as “*man is to king as woman is to queen*” [11]. Examples for health care include the following:

- “*'acetaminophen' is as type of 'drug' as 'diabetes' is as type of ‘disease’*” [12].
- “*(furosemide - kidney) + heart ~ fosinopril*” [13].

This study aimed to investigate embedding analogies (analogical reasoning with embeddings) [5] that are not pair-based proportional (pairwise for short) analogies. This study began by observing senior clinicians performing an analogical reasoning for sepsis (a major life-threatening condition) with embeddings and posing search queries such as *−sepsis +serum_albumin +fluid_therapy* to discover treatments with therapeutic intent. The clinical rationale behind this query is that “*current evidence suggests that resuscitation using albumin-containing solutions is safe*” [14], where *serum_albumin* is a shortened form of “*human serum albumin supplementation*” (extensively debated for sepsis [15]). We viewed this as another type of *the four-term analogy,* which is not pairwise.

This paper presents a semiautomatic approach to extract meaning (semantics) from the unstructured free text of biomedical literature (ie, PubMed systematic reviews [16]). The disease treatment statements systematically acquired from analogical reasoning are biomedical facts validated with evidence first and human audit afterward. The approach presented belongs to Semantic Deep Learning (SemDeep) [17], as we used embedding analogies (other than pairwise) and semantic knowledge representation paradigms [18] to provide meaning for the same.

### Analogical Reasoning

Humans possess the ability to reason by analogy using abstract semantic relations such as synonyms or category membership [3]. For example, *common cold* and *influenza* are both types of illnesses with some common symptoms such as runny nose, sore throat, cough, and headache. As they share some key characteristics, we can possibly say they are near-synonyms, although they cannot be used interchangeably (as synonyms would) because of key medical differences. Our SemDeep approach acquires terms about treatments for a well-known disease using analogical reasoning that is underpinned by Aristotle’s theory [19]:

- “*The strength of an analogy depends upon the number of similarities*” [19]. For example, “intravenous antibiotics” and “intravenous fluid resuscitation” are basic therapies that improve outcomes in patients with sepsis [14], that is, both are treatments with a therapeutic intent for sepsis. However, we cannot say that they are similar as “intravenous fluid resuscitation” is a procedure whereas an “intravenous antibiotic” is a substance, although both are “intravenous.”
- “*Similarity reduces to identical properties and relations*” [19]. For example, “benzyl penicillin,” “cefotaxime,” or “amoxicillin/clavulanate” is similar as they belong to the same category, “antipseudomonal beta-lactam antibiotics” [14].
- "*Good analogies derive from underlying common causes or general laws*” [19]. This study investigated the systematic acquisition of treatments for a disease using the simple generic 3CosAdd formula [20,21].

### The 3CosAdd Formula

Our work relied on vector semantics [5] and used the neural language models, Continuous Bag-of-Words (CBOW) and Skip-gram by Mikolov et al [20], from deep learning to create embeddings. Embeddings with vector semantics such as cosine or 3CosAdd can acquire a list of strings of characters (eg, n-grams), although they lack explicit semantic meaning. Until now, the 3CosAdd formula 1 [20,21] has been applied to analogies between 2 pairs of words a:a*::b:b* [7], where b* is the unknown (hidden) word.

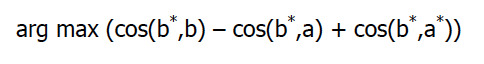



We used the 3CosAdd formula as in Levy and Goldberg [21] with the rewording “*find the term y, which is similar to the term z1 and the term z2, while different from the term x*”, where the target term *x* provides the semantic context and similar refers to the terms sharing “commonalities in structural features.” In this study, a semantic field is a set of terms that “*belong together under the same conceptual heading*” [22] and is a form of knowledge representation that provides meaning to those terms. We rewrote the 3CosAdd formula as formula 2, with the search query *-x +z1 +z2*, and the type of 4-term analogy we sought had the following:

- The target term *x* belonging to the semantic field *disease* and representing a medical diagnosis mappable to a “type of” of systematized nomenclature of medicine - clinical terms (SNOMED CT) [23] concept called disorder.
- The 3 terms {*z1; z2; y*} belonging to the semantic field *treatment*(Tx for short), where Tx encompasses 3 textual definitions from Hart et al [24]. The candidate term *y* is the unknown.

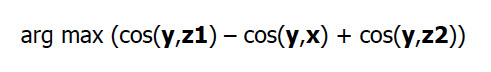

### The Research Questions

We adopted the view by Hill et al [25] by considering “relatedness” as “association” and synonymy as the strongest similarity. In this study, the association relationship of interest is “correlation,” as defined in the semantic science integrated ontology (SIO) [26].

As preliminaries, we asked 2 research questions not specific to the health care domain:

- Q1: Can “good” embeddings be created with a small corpus?
- Q2: If the simple generic 3CosAdd formula [20,21] can capture a type of 4-term analogy as read in formula 2, can they be observed in embeddings created with a small corpus?

Our third research question (Q3) asked whether the 4-term type of analogy discovered in a small common-English corpus can also be discovered in a larger-scale biomedical corpus. To provide proof of such a generalization, we performed a real-world test with embeddings created with free text from PubMed systematic reviews [16]. We postulated that candidate inferences can be validated using evidence-based information resources. This study investigated the discovery of *clinical winners*, that is, search queries *-x +z1 +z2* bringing candidate treatments *y* with evidence of a therapeutic intent for target disease *x*; thus, enabling the most common type of clinical question, “how to treat a disease or condition” [1], to be answered.

Our final research question (Q4) asked for some predictive power over the *clinical winners* obtained (ie, an empirical heuristic) if our SemDeep approach worked, that is the type of analogy proposed finds disease treatment statements from PubMed systematic reviews (ie, a larger-scale biomedical corpus). This last question pursued a tacit preference and referred to the final characteristic of analogy: systematicity [6]. However, challenges have been acknowledged “for any vector space model that aims to make predictions about relational similarity” [27].

Between the semantic field *disease* and the semantic field *treatment*, “*few maximal structurally consistent interpretations (ie, mappings displaying one-to-one correspondences and parallel connectivity)*” [6] are to be expected. For example, aspirin treatment does not have a one-to-one correspondence with a disease as it can treat headache (common knowledge) and acute myocardial infarction [1]. In this study, “spontaneous unplanned inferences” [6] were also expected, and this propensity was captured with the notion of incremental mappings [6].

## Methods

### Overview

**Figure 1:**
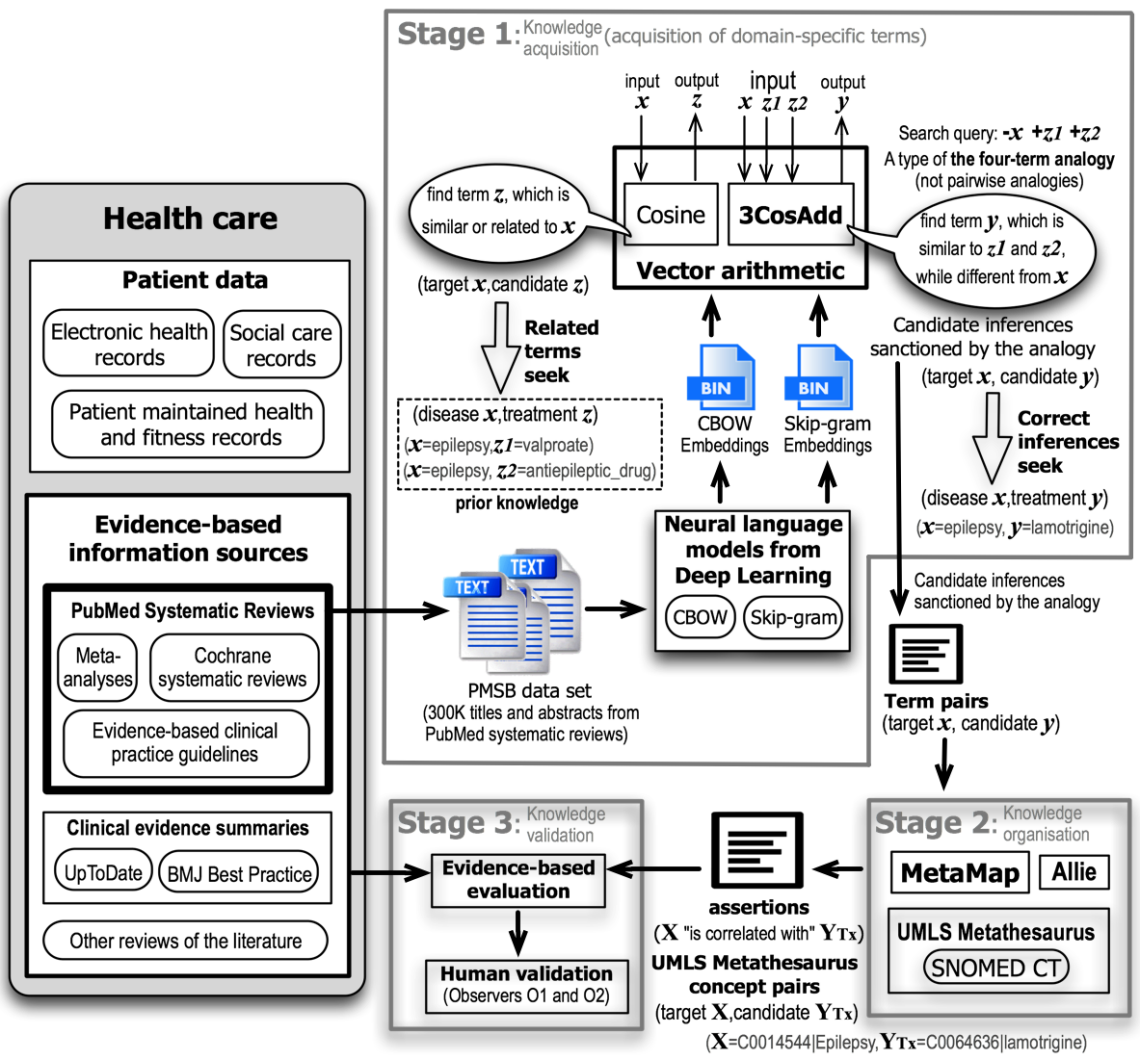
Overview of our SemDeep approach.

Our SemDeep approach answered Q3 and comprised the 3 stages depicted in Figure 1. The software package word2vec [28] implements the CBOW and Skip-gram algorithms along with the cosine and 3CosAdd formulas. The terms in this study are n-grams.

Stage 1 used prior knowledge (open-access reusable datasets [29]) consisting of n-gram pairs obtained by applying the cosine to embeddings, then mapped to the Unified Medical Language System (UMLS) Metathesaurus [30] concept pairs, and finally validated with evidence from biomedical literature using the British Medical Journal (BMJ) Best Practice [31] as the main information source.

BMJ Best Practice is separate from PubMed/MEDLINE [32] and is acknowledged for its editorial quality and evidence-based methodology [33]. In the United Kingdom, BMJ Best Practice is provided (free access) to all National Health Service (NHS) health care professionals in England, Scotland, and Wales [34]. BMJ Best Practice provides advice on symptom evaluation, tests to order, and treatment approach structured around the patient consultation.

We started by investigating embedding analogies in a small common-English corpus to answer Q1 and Q2.

### Preliminaries: Analogies for Shakespeare’s Romeo in a Small Common-English Corpus

Topic models are related to semantic fields [5]. There are many small corpora and tutorials illustrating the inner workings of topic models, such as the spatially motivated Latent Semantic Analysis (LSA) method [35] and the probabilistic method latent Dirichlet allocation (LDA) [36]. We used a small common-English corpus appearing in an LSA tutorial [37]. Textbox 1 shows the corpus used to answer Q1 and Q2.

**A small common-English corpus consisting of 5 lines of text.**

Romeo and Juliet

Juliet: Oh happy dagger!

Romeo died by dagger.

“Live free or die”, that’s the New-Hampshire’s motto

Did you know New-Hampshire is in New-England?

In common English, punctuation marks can change the meaning of a sentence. For example, “prevail, not perish” versus “prevail not, perish.” We did not transform routine letters into lowercase letters and did not remove punctuation marks, with the only exception of double quotations. Multimedia Appendix 1 contains the input text and the hyperparameter configuration for the CBOW model with word2vec [28].

Below, we summarize the answers to Q1 and Q2 (Multimedia Appendix 1):

- Answer to Q1: A “good” vector semantic model should find a candidate *y* that is “semantically similar” to the target *x = Romeo*. The candidate *y* with the highest cosine for the CBOW model is *you*: The terms *you* and *Romeo* are near-synonyms, that is “interchangeable in some contexts” [38]. Hence, the answer to Q1 is “yes.”
- Answer to Q2: We applied the 3CosAdd formula 2, where the target *x = Romeo* provides the semantic context. The terms *die = z1* and *died = z2* from the corpus are representative of inflectional morphology *infinitive:past*. The search query *–x +z1 +z2* is posed to the CBOW model, that is “find the term *y*, which is similar to *die* and *died*, while different from *Romeo*”. Candidate *y* with the highest 3CosAdd is “*dagger*.” The term *dagger* belongs to the semantic field *death* as “dagger is an instrument that causes death”; thus, the candidate inference is true. Hence, the answer to Q2 is also “yes.”

#### Stage 1: Knowledge Acquisition (Acquisition of Domain-Specific Terms)

The PubMed systematic reviews (in Figure 1) [16] is an evidence-based searching filter “AND (systematic [sb])”, intended for retrieving “best evidence” information sources from PubMed/MEDLINE [32] such as Cochrane systematic reviews [39]. Health care–related institutions such as the World Health Organization promote PubMed searches with this filter (examples in Prevention and Control of Noncommunicable Diseases: Guidelines for Primary Health Care in Low Resource Settings [40]).

This study used a subset of PubMed systematic reviews [16] of 301,201 PubMed/MEDLINE publications (titles and available abstracts), called the PubMed systematic reviews subset (PMSB dataset). The preprocessing of the input text for the PMSB dataset and the hyperparameter configuration for Skip-gram and CBOW are identical to those in our previous study [41] and detailed in the study by Arguello Casteleiro et al [42].

From the PMSB dataset, a total of 423K n-grams with a frequency count >5 have vector representations in both models, that is CBOW and Skip-gram. We considered “good” the Skip-gram and CBOW embeddings created in our previous study [41] as they both perform well (using conventional evaluation measure precision [43]) in semantic similarity and relatedness tasks with the cosine formula. The n-gram *z* reused in this study (ie, z1 and z2) is from our previous study [41].

### Applying the 3CosAdd Formula to Acquire the Top 12 Ranked Term Pairs (x,y): A 4-Term Analogy

To address Q3 and apply the 3CosAdd formula 2, 2 n-gram pairs (disease *x*,treatment *z*) from our previous study (prior knowledge) [41] were needed. We kept only the 12 top-ranked candidate n-grams *y* for the 3CosAdd formula, that is, the 12-candidate *y* with CBOW and Skip-gram embeddings yielding the highest 3CosAdd values. We limited the list of candidates to 12, similar to Arguello Casteleiro et al [42], and following cognitive theories like Novak JD and Cañas AJ [44].

#### Stage 2: Knowledge Organization (Explicit Conceptualization of the Meaning of Terms)

This stage accomplishes a named entity recognition (NER) [45] task involving 3 domain experts (2 biomedical terminologists and 1 medical consultant who performs clinical coding). Every UMLS Metathesaurus concept has a concept unique identifier (CUI) and at least one UMLS Semantic Type (broad category) [30] assigned. The NER task consists of 3 sequential subtasks (Multimedia Appendix 1):

- First, disambiguation of n-grams *y* is difficult to interpret for being truncated strings of characters or containing short forms (eg, abbreviations or acronyms). String searches in the PMSB dataset and the web search the sense inventory, Allie [46], enabling disambiguation.
- Second, the manual binary classification of candidate n-gram *y* as to whether it belongs to the semantic field Tx (ie, *y_Tx_*). Following Artstein R and Poesio M [47], we reported the interrater agreement with a Krippendorff alpha [48].
- Third, entity normalization (grounding) [49] with MetaMap [50], where 3 domain experts apply the NER guidelines for MetaMap's output [51] and together judge the automatic mapping of n-grams *y_Tx_* to UMLS Metathesaurus concepts *Y_Tx_*. MetaMap performance is calculated using precision, recall, and F-measure [43,52].

We took n1 as the number of different UMLS Metathesaurus concepts (represented as Z1 and Z2) mapped as *z1* and *z2* in the search query. Once the NER task was completed, we obtained the *NER winners*. An *NER winner* was a search query *-x +z1 +z2* with the maximum observed number for n2 or n3:

- n2 is the number of different 12 top-ranked candidate n-grams *y* belonging to Tx, that is, the number of *y_Tx_*.
- n3 is the number of different UMLS Metathesaurus concepts *Y_Tx_* excluding Z1 and Z2.

#### Stage 3: Knowledge Validation (Validating Statements)

We sought evidence for the Metathesaurus concept pairs (*X,Y_Tx_*) acquired previously to determine the therapeutic intent of candidate *Y_Tx_* for target disease *X*, where *X* was the UMLS Metathesaurus concept mapped to n-gram *x*.

The same 3 domain experts from Stage 2 triaged the results of manual literature searches considering the following:

1. The type of evidence-based information sources, seeking the “best evidence.” Evidence-based medicine [53] categorizes and ranks different types of clinical evidence [1]. For example, the Cochrane systematic reviews are at the forefront of “best evidence” [1], whereas studies of the physiological functions and clinicians’ observations are considered evidence of least value [1].
2. The publication date, seeking the “most recent papers published.”

The 3 domain experts introduced 6 evidence-based categories to further refine the correlations between the semantic field *disease* and *treatment* (Tx). Table 1 illustrates them with examples of evidence (quoted text) and references for the UMLS Metathesaurus concepts *Y_Tx_* related to the target concept disease *X*=“C024302|Sepsis” with CUI=C024302. The rationale for the 7 evidence-based categories introduced is as follows:

- The name of 4 of the evidence-based categories (top rows in Table 1) resembles the categories “beneficial, likely beneficial, no known benefit, harmful” for health care interventions from the decommissioned BMJ Clinical Evidence (predecessor of BMJ Best Practice [31]).
- The evidence-based category “Tx ingredient” acknowledges that a complex treatment may have parts, that is “partitive relationships” [54].
- The evidence-based category “correlation” captures “spontaneous unplanned inferences” [6].
- The evidence-based category “general medical term” includes broad concepts of little value for clinicians that do not need further evidence (quotes and references).

This study distinguishes between *NER winners* (maximum observed number for n2 or n3 in Stage 2) and *clinical winners*. A *clinical winner* is a search query *-x +z1 +z2* (a type of 4-term analogy) for target disease *x* with a maximum observed value for n4, that is, the number of different concepts *Y_Tx_* (excluding Z1 and Z2) assigned to the evidence-based category “Tx with therapeutic effect.”

To audit the evidence-based categories assigned along with the evidence collected (quotes and references) for the concept pairs (*X,Y_Tx_*), 2 more observers (O1 a medical consultant and O2 a BMJ health informatician who works with BMJ Best Practice content and has a junior doctor background) were asked to express agreement or disagreement with the evidence for the concept pairs (*X,Y_Tx_*). Multimedia Appendix 1 has the evaluation guidelines given to the observers. Cohen kappa [55] was used to measure interobserver agreement.

**Table 1:** Evidence from the literature searches, that is quoted text and reference, for unified medical language system Metathesaurus concept pairs (X, Y_Tx_) with X=C0243026|Sepsis.

Candidate concept *Y*_*Tx*_; UMLS CUI|Concept name^a^	Evidence-based categories for concept *Y*_*Tx*_ correlated with concept *X*	Evidence (quoted text) [evidence source] [citation]
C0056562|crystalloid solutions	Tx with therapeutic effect	“Step-by-step treatment approach: ... Administer 30 mL/kg crystalloid for hypotension or lactate ≥4 mmol/L (≥36 mg/dL)” [BMJ BP topic: 245] [14]
C0001617|Adrenal Cortex Hormones	Tx with uncertain therapeutic effect	“Step-by-step treatment approach: Adjunctive therapies ... evidence for giving corticosteroids to patients with sepsis or septic shock is mixed.” [BMJ BP topic: 245] [14]
C0020352|Hetastarch	Tx with unwanted or adverse effects (ie, nontherapeutic)	“Step-by-step treatment approach: Fluid resuscitation ... HES solutions for infusion have been significantly restricted across the European Union and are contraindicated in critically ill patients and those with sepsis or renal impairment.” [BMJ BP topic: 245] [14]
C0677850|Adjuvant therapy	Potential Tx (under research and development)	“Adjuvant immune therapy to manipulate the hyper-inflammatory and/or immune-suppressive phase of sepsis is an attractive therapeutic option, which may improve outcome and ease the burden of antimicrobial resistance. However, before this can become a clinical reality, we must recognise that sepsis is a clinical syndrome, where significant heterogeneity exists.” [PMID: 30515242] [56]
C3273371|CD4 Positive Memory T-Lymphocyte	Tx ingredient	“Administration of immune-modulatory therapy is a promising treatment approach for treating sepsis survivors. … these therapies can improve pathogen clearance, increase CD4 T cell responsiveness, and promote survival in sepsis.” [PMID: 24791959] [57]
C0745442|Intravenous Catheters	Tx ingredient	“Recommendations: Monitoring ... Central venous catheters will be required to ensure reliable delivery of vasoactive medication.” [BMJ BP topic: 245] [14]
C0812144|Medication administration: epidural	Correlation (epidural → potential sites of infection: epidural sites → sepsis: investigations)	“Investigations to identify causative organisms: ... If no localising signs are present, examination and culture of all potential sites of infection including wounds, catheters, prosthetic implants, epidural sites, and pleural or peritoneal fluid, as indicated by the clinical presentation and history, is required.” [BMJ BP topic: 245] [14]
C0013227|Pharmaceutical Preparations	General medical term	—^b^

^a^The references shown are either the PubMed identifier (PMID) or the topic number in BMJ Best Practice (“BMJ BP topic” for short).

^b^The evidence-based category “general medical term” has no evidence (quoted text).

## Results

We obtained 5352 n-gram pairs from 446 search queries by applying the 3CosAdd formula and taking the top 12 values. These are presented in Multimedia Appendix 2 (worksheet Stage 1). These n-gram pairs are enriched with domain knowledge meaning (Stage 2) and the biomedical evidence found from literature searches is ratified with an audit (Stage 3).

### Stage 1: Knowledge Acquisition (Acquisition of Domain-Specific Terms)

To apply the 3CosAdd formula (and systematic creation of search queries), we reused 63 unique n-gram pairs (*x,z*) from our previous study [41] (open-access [29]). Every reused n-gram *z* was mapped to the UMLS concept *Z* with the UMLS Semantic Type “T061|Therapeutic or Preventive Procedure” or “T121|Pharmacologic Substance.” Multimedia Appendix 1 has the UMLS CUI pairs (*X,Z*).

#### Applying the 3CosAdd Formula to Acquire the Top 12 Ranked Term Pairs (x,y): A 4-Term Analogy

With 63 n-gram pairs (*x,z*), we built 223 search queries *-x +z1 +z2* for the 3CosAdd formula. Multimedia Appendix 2 (worksheet Stage 1) contains the 223 search queries and the 5352 (*x,y*) n-gram pairs for 10 target diseases *x*, that is, the 12 top-ranked n-grams (highest 3CosAdd value) obtained per search query from the CBOW and Skip-gram embeddings. An n-gram pair with *y* as a non-ASCII character was discarded.

### Stage 2: Knowledge Organization (Explicit Conceptualization of the Meaning of Terms)

Different search queries brought the same (target *x*,candidate *y*) n-gram pairs from applying the 3CosAdd formula. Multimedia Appendix 2 (worksheet Stage 2) has 1935 unique (*x,y*) n-gram pairs from the 5352 n-gram pairs. Among the 1935 unique (*x,y*) n-gram pairs, there were 954 n-gram pairs (*x,y_Tx_*) with candidate *y* belonging to Tx. The Krippendorff alpha [48] was 0.86 for the 3 domain experts for the binary classification (Tx or non-Tx). Considering all candidates *y_Tx_* mapped to *Y_Tx_* for the 10 diseases (microaveraging) [43], MetaMap had an F-measure=80.00% with precision=77.00% and recall=83.25%. Multimedia Appendix 1 has the detailed results for NER subtasks, including an investigation of the UMLS semantic types for *Y_Tx_*.

Table 2 contains the *NER winners*, that is, the search query *-x +z1 +z2* for the 3CosAdd formula per model and disease target *x* having the maximum observed values for n2 or n3.

- The maximum observed value for n2 was the highest possible value, that is, n2=12, for both CBOW and Skip-gram.
- The maximum observed value for n3 was for the search query, *−epilepsy +valproate +AED*. However, the number of different *Y_Tx_* (excluding Z1 and Z2) differed, that is, n3=11 for Skip-gram and n3=10 for CBOW.

### Stage 3: Knowledge Validation (Validating Statements)

Multimedia Appendix 2 (worksheet Stage 3) has the 569 unique UMLS Metathesaurus concept pairs (*X,Y_Tx_*) mapped to the unique 954 n-gram pairs (*x,y_Tx_*). Although the UMLS related concepts table (file=MRREL) [58] contains relationships asserted by source vocabularies between CUI pairs, only 68 of the 569 CUI pairs appeared within the MRREL table of 2019AA UMLS release.

Manual searches in the literature proved to be time-consuming and labor-intensive; thus, not all the concept pairs for the target disease anemia and hypertension had evidence. Hence, we limited the study to 408 UMLS CUI pairs (Multimedia Appendix 1), and only 59 of these were within the MRREL table (column J of Multimedia Appendix 2 worksheet Stage 3).

**Table 2:** NER winners per target disease x (search query -x +z1 +z2) for the 3CosAdd formula, that is, the highest value for n2 or n3 per model and per disease target x.

Disease target *x*	Model	NER max (n2)	NER max (n3)	Treatment *z1* search query	Treatment *z2* search query	n1	n2	n3
heart_failure	CBOW^a^	Yes	N/A^b^	angiotensin-converting_enzyme_(ACE)_inhibitors	aldosterone_antagonists	2	12	6
heart_failure	CBOW	N/A	Yes	cardiac_resynchronization_therapy_(CRT)	aldosterone_antagonists	2	10	9
heart_failure	Skip-gram	Yes	Yes	beta-blockers	aldosterone_antagonists	2	12	8
glaucoma	CBOW	Yes	Yes	trabeculectomy	cataract_surgery	2	5	5
glaucoma	Skip-gram	Yes	Yes	trabeculectomy	cataract_surgery	2	10	6
CKD^c^	CBOW	Yes	Yes	not_requiring_dialysis	dialysis	1	9	7
CKD	Skip-gram	Yes	Yes	not_requiring_dialysis	dialysis	1	8	5
diabetes	CBOW	Yes	Yes	glucose_variability	glucagon-like_peptide-1_receptor_agonists	2	10	6
diabetes	Skip-gram	Yes	Yes	glucose_variability	glucagon-like_peptide-1_receptor_agonists	2	10	5
asthma	CBOW	Yes	N/A	inhaled_corticosteroid	LABAs^d^	2	11	6
asthma	CBOW	N/A	Yes	inhaled_corticosteroids	inhaled_corticosteroid	1	10	8
asthma	Skip-gram	Yes	Yes	anti-LTs	LABAs	2	12	8
epilepsy	CBOW	Yes	Yes	valproate	AED^e^	2	12	10
epilepsy	Skip-gram	Yes	Yes	valproate	AED	2	12	11
arthritis	CBOW	Yes	Yes	plus_methotrexate	methotrexate	1	12	9
arthritis	Skip-gram	Yes	Yes	methotrexate	DMARDs^f^	2	11	6
osteoarthritis	CBOW	N/A	Yes	hyaluronic_acid	glucosamine	2	8	9
osteoarthritis	CBOW	Yes	N/A	knee_arthroplasty	hyaluronic_acid	2	9	7
osteoarthritis	CBOW	N/A	Yes	vs_acetaminophen	glucosamine	2	8	9
osteoarthritis	Skip-gram	Yes	Yes	vs_acetaminophen	hyaluronic_acid	2	11	8
anaemia	CBOW	Yes	Yes	iron	erythropoiesis-stimulating_agents	2	11	9
anaemia	Skip-gram	Yes	N/A	blood_transfusions	ESAs^g^	2	12	6
anaemia	Skip-gram	N/A	Yes	recombinant_human_erythropoietin	iron	2	11	8
hypertension	CBOW	Yes	N/A	antihypertensive_drugs	angiotensin_receptor_blockers	2	12	6
hypertension	CBOW	N/A	Yes	antihypertensive_therapy	antihypertensive	2	11	8
hypertension	Skip-gram	Yes	Yes	antihypertensive_drug_classes	antihypertensive	1	12	10

^a^CBOW: Continuous Bag-of-Words.

^b^N/A: not applicable.

^c^CKD: chronic kidney disease.

^d^LABA: long-acting beta2-agonist.

^e^AED: antiepileptic drug.

^f^DMARD: disease-modifying antirheumatic drug.

^g^ESA: erythropoiesis-stimulating agent.

Table 3 shows the 7 evidence-based categories assigned to the 408 UMLS CUI pairs investigated thoroughly. There are 19 concept pairs (*X,Y_Tx_*) with more than 1 evidence-based category, such as the concept pair (*X*=C0014544|Epilepsy,*Y_Tx_*=C0080356|Valproate). The evidence-based category “Tx with therapeutic effect” has the highest number of CUI pairs, with 190 pairs (*X,Y_Tx_*), where 117 pairs have evidence (quotes) taken from BMJ Best Practice. The evidence-based category “correlation” has the highest number of evidence-based information sources with 108 uniform resource identifiers of the total 238. Multimedia Appendix 1 has further details.

Table 4 shows the *clinical winners*, that is, search query -x +z1 +z2 (a type of 4-term analogy) with the maximum observed number for n4 per target disease x. Table 4 reveals that an *NER winner* is not necessarily a *clinical winner*, that is, the maximum observed value for n4 does not always correspond to the maximum observed value for n3 or n2.

**Table 3:** The 408 unified medical language system concept unique identifier pairs investigated thoroughly and their evidence-based information sources per evidence-based category.

Evidence-based categories for concept Y_Tx_ correlated with concept X	Number of CUI^a^ pairs	Number of evidence-based information sources (ie, URIs^b^) for CUI pairs	Number of CUI pairs with BMJ Best Practice as evidence source
Tx with therapeutic effect	190	73	117
Tx with uncertain therapeutic effect	38	22	11
Tx with unwanted or adverse effects (ie, nontherapeutic)	52	41	17
Potential Tx (under research and development)	5	5	0
Tx ingredient	22	21	6
General medical term	26	0	0
Correlation	94	108	19

^a^CUI: concept unique identifier.

^b^URI: Universal Resource Identifier.

In Table 4, there are two rows that are not *clinical winners* according to the observer O2. All rows except two are *clinical winners* according to the 3 domain experts and both observers.

Considering the 408 concept pairs (*X,Y_Tx_*) with evidence, observer O1 disagrees with 25 of them, and observer O2 disagrees with 26 of them. The Cohen kappa of −0.023 is paradoxical [59-61], resolved in Multimedia Appendix 1 following Cicchetti DV and Feinstein AR [61].

Table 5 shows how the evidence-based category “Tx with therapeutic effect” assigned by an observer (when in disagreement) affects the *clinical winners* from Table 4. For observer O1, the only change was a decrease of n4 from 5 (Table 4) to 4 (Table 5) in the search query, *−anaemia +recombinant_human_erythropoietin +iron*, for Skip-gram. The observer O2 provided additional therapeutic evidence from BMJ Best Practice when in disagreement, typically increasing n4 or making “new” *clinical winner*s (eg, search query, *−epilepsy +valproate +levetiracetam*).

**Table 4:** Clinical winners (highest value of n4) per model and disease target x considering the 3 domain experts.

Disease target *x*	Model	NER max (n2)	NER max (n3)	Treatment *z1* search query	Treatment *z2* search query	n1	n2	n3	n4
heart_failure	CBOW^a^	—	Yes	cardiac_resynchronization_therapy_(CRT)	aldosterone_antagonists	2	10	9	6
heart_failure	Skip-gram	Yes	Yes	beta-blockers	aldosterone_antagonists	2	12	8	5
glaucoma	CBOW	Yes	Yes	trabeculectomy	cataract_surgery	2	5	5	3
glaucoma	Skip-gram	Yes	Yes	trabeculectomy	cataract_surgery	2	10	6	3
CKD^b^	CBOW	Yes	Yes	not_requiring_dialysis	dialysis	1	9	7	5
CKD	Skip-gram	Yes	Yes	not_requiring_dialysis	dialysis	1	8	5	5
diabetes	CBOW	Yes	Yes	glucose_variability	glucagon-like_peptide-1_receptor_agonists	2	10	6	6
diabetes	Skip-gram	Yes	Yes	glucose_variability	glucagon-like_peptide-1_receptor_agonists	2	10	5	4
asthma	CBOW	—	Yes	inhaled_corticosteroids	inhaled_corticosteroid	1	10	8	8
asthma	Skip-gram	—	—	inhaled_corticosteroids	inhaled_corticosteroid	1	11	8	7
epilepsy	CBOW	—	—	valproate	antiepileptic_drug	2	11	10	8
epilepsy	CBOW	—	—	valproate	antiepileptic_drugs	2	11	10	8
epilepsy^c^	Skip-gram	Yes	Yes	valproate	AED^d^	2	12	11	7
arthritis	CBOW	Yes	Yes	plus_methotrexate	methotrexate	1	12	9	2
arthritis^c^	Skip-gram	—	—	plus_methotrexate	methotrexate	1	7	4	2
osteoarthritis	CBOW	Yes	—	knee_arthroplasty	hyaluronic_acid	2	9	7	5
osteoarthritis	Skip-gram	—	—	vs_acetaminophen	viscosupplementation	2	7	8	7
anaemia	CBOW	Yes	Yes	iron	erythropoiesis-stimulating_agents	2	11	9	4
anaemia	Skip-gram	—	Yes	recombinant_human_erythropoietin	iron	2	11	8	5
hypertension	CBOW	—	Yes	antihypertensive_therapy	antihypertensive	2	11	8	6
hypertension	Skip-gram	Yes	Yes	antihypertensive_drug_classes	antihypertensive	1	12	10	8

^a^CBOW: Continuous Bag-of-Words.

^b^CKD: chronic kidney disease.

^c^Not *clinical winners* according to O2.

^d^AED: antiepileptic drug.

**Table 5:** Changes in clinical winners (highest value of n4) per model and disease target x considering observer O1 and O2.

Disease target *x*	Model	Differences in *clinical winner* max (n4) according to observers	Treatment *z1* search query	Treatment *z2* search query	n1	n2	n3	n4
epilepsy	Skip-gram	Observer O2^a^: New	valproate	levetiracetam	2	10	9	7
arthritis	CBOW^b^	Observer O2: n4 different	plus_methotrexate	methotrexate	1	12	9	6
arthritis	CBOW	Observer O2: New	methotrexate	DMARDs^c^	2	11	9	6
arthritis	Skip-gram	Observer O2: New	IACI^d^	DMARDs	2	9	6	5
arthritis	Skip-gram	Observer O2: New	plus_methotrexate	DMARDs	2	10	6	5
anaemia	Skip-gram	Observer O1^e^: n4 different	recombinant_human_erythropoietin	iron	2	11	8	4

^a^O2: BMJ health informatician who works with BMJ Best Practice content and has a junior doctor background.

^b^CBOW: Continuous Bag-of-Words.

^c^DMARD: disease-modifying antirheumatic drug.

^d^IACI: intra-articular corticosteroid injection.

^e^O1: medical consultant.

Multimedia Appendix 1 has the best *clinical winner,* which is an *NER winner*. Table 6 shows the best *clinical winner* that is not an *NER winner*. Table 6 illustrates the enrichment of the candidate n-grams *y* with domain knowledge meaning (Stage 2 normalizes n-grams with UMLS CUIs) and biomedical evidence ratified with an audit (Stage 3). The evidence provided for the evidence-based categories (quotes with references from the biomedical literature) is presented in Multimedia Appendix 2 (worksheet Stage 3).

In conclusion, considering the *clinical winners* found (Table 4), the answer to Q3 is “yes,” that is, the 4-term type of analogies discovered in a small common-English corpus can also be discovered in a large-scale biomedical corpus.

**Table 6:** Illustration of a Best clinical winner with max (n4)=8 for CBOW and disease target x = epilepsy, which is not an NER winner.

Rank^a^	Candidate *y*	3CosAdd	UMLS CUI for concept *Y*_*Tx*_ mapped to candidate *y*_*Tx*_	Evidence-based categories for concept *Y*_*Tx*_ correlated with concept *X*
1	lamotrigine	0.385201	C0064636	Tx with therapeutic effect
2	carbamazepine	0.345227	C0006949	Tx with unwanted or adverse effects (ie, nontherapeutic)
3	low_propensity	0.324285	—^b^	—
4	clonazepam	0.310706	C0009011	Tx with uncertain therapeutic effect
5	topiramate	0.308402	C0076829	Tx with therapeutic effect
6	lithium_valproate	0.308223	C0023870|C0080356	Tx with therapeutic effect|Tx with unwanted or adverse effects (ie, nontherapeutic)
7	clobazam	0.306901	C0055891	Tx with therapeutic effect
8	sodium_valproate	0.300513	C0037567	Tx with therapeutic effect
9	lorazepam	0.29562	C0024002	Tx with therapeutic effect
10	lithium	0.294804	C0023870	Tx with therapeutic effect|Tx with unwanted or adverse effects (ie nontherapeutic)
11	gabapentin_pregabalin_topiramate	0.291698	C0657912|C0076829|C0060926	Tx with therapeutic effect
12	antiepileptic_drugs_other_than	0.290046	C0003299	Tx with therapeutic effect

^a^The search query −x +z1 +z2 is listed in Table 4, which is −epilepsy +valproate +antiepileptic_drug. The character “|” appears when there is more than 1 CUI or evidence-based category.

^b^The candidate y = “low_propensity” does not belong to the semantic field Tx, and so, it has no UMLS CUI assigned.

### Answer Q4: An Empirical Heuristic with Some Predictive Power for Clinical Winners

Multimedia Appendix 2 (worksheet Q4) has the 304 search queries of the total of 446 (223 for CBOW and 223 for Skip-gram) queries, where all the candidates *y_Tx_* mapped to concepts *Y_Tx_* have at least one evidence-based category assigned. Textbox 2 summarizes the empirical heuristic developed by visual inspection, focusing on rows with the minimum (n4=0) and the maximum observed values of n4. The heuristic is programmatically implemented as a Boolean expression composed of 3 expressions with the Boolean AND.

**An empirical heuristic developed by visual inspection with some predictive power for the clinical winners.**

1. Avoid n-grams *z1* and *z2* having short forms
2. Favor n-grams *z1* or *z2* (or both) not appearing among the 20 top-ranked candidates for target *x* with the highest value for cosine with Skip-gram embeddings
3. Favor n-gram *z2* with frequency counts in the corpus >100

The heuristic selects 93 of the 304 search queries, which brings 126 of the 190 UMLS Metathesaurus concepts *Y_Tx_* with the evidence-based category “Tx with therapeutic effect,” that is, *Y_Tx_* with therapeutic intent.

Table 7 (source data in Multimedia Appendix 1) shows the performance of the heuristic considering (1) the values of n4 (the last 3 yellow columns in Multimedia Appendix 2 worksheet Q4), (2) the different thresholds for n4, and (3) precision and recall as metric.

Considering the precision and recall values for the empirical heuristic (Table 7), the answer to Q4 is also “yes,” that is, some predictive power over the *clinical winners* obtained is possible.

**Table 7:** Precision and recall for the empirical heuristic developed using Multimedia Appendix 2 (worksheet Q4).

Threshold	True positive (TP)	False positive (FP)	False negative (FN)	Precision^a^ %	Recall^b^ %
n4>0	91	2	189	97.85	32.5
n4>1	84	9	151	90.32	35.74
n4>2	73	20	111	78.49	39.67
n4>3	48	45	76	51.61	38.71
n4>4	28	65	52	30.11	35
n4>5	14	79	20	15.05	41.18
n4>6	9	84	5	9.68	64.29
n4>7	4	89	0	4.3	100

^a^Precision: calculated as TP/(TP+FP).

^b^Recall: calculated as TP/(TP+FN).

## Discussion

### Principal Findings

Humans can agree that the semantic field *person* {you; Romeo} is related to the semantic field *death* {die; died; dagger} in the context of Shakespeare’s Romeo. Hence, we answer Q1 and Q2 with a “yes”; therefore, analogical reasoning with CBOW embeddings seems feasible with a small common-English corpus. This challenges the current assumption that “learning in current deep learning models relies on massive data” [3].

We answered Q3 by demonstrating that there is proof of the generalization; thus, the 3CosAdd formula can discover another type of 4-term analogy that is not a pair-based proportional analogy. Furthermore, we have proven that the analogical inferences sanctioned by the 3CosAdd formula with embeddings could extract treatments with therapeutic intent from free text. Indeed, there were strong examples of analogical reasoning with abstract semantic relations between *z1* and *z2* among *clinical winners* (Table 4):

- *Antonym.* The search query, *−CKD +not_requiring_dialysis +dialysis*, with n4=5 for CBOW and Skip-gram.
- *Synonym.* The search query, *−asthma +inhaled_corticosteroids +inhaled_corticosteroid*, with n4=8 for CBOW (the best *clinical winner*) and n4=7 for Skip-gram, where the relation between *z2* and *z1* was inflectional morphology *singular:plural*. This query resembled the search query, *−Romeo +die +died*.
- *Category membership*. The search query, *−epilepsy +valproate +antiepileptic_drug*, with n4=8 for CBOW. The search query, *−hypertension +antihypertensive_drug_classes +antihypertensive*, with n4=8 for Skip-gram. Both search queries were the best *clinical winners* (maximum observed value for n4).
- *Commonalities in structural features*. All search queries focused on the therapeutic intent of *z1* and *z2* for target disease *x*. However, some queries did not have the above abstract semantic relationships between *z1* and *z2*. For example, the search queries *−osteoarthritis +knee_arthroplasty +hyaluronic_acid* with n4=5 for CBOW and *−heart_failure +beta-blockers +aldosterone_antagonists* with n4=5 for Skip-gram.

We answered Q4 by demonstrating that it is feasible to gain some predictive power for the *clinical winners*; therefore, a tactic preference was latent promising systematicity [6]. Textbox 3 highlights the precision and recall values for 3 n4 thresholds of the overall performance of the empirical heuristic developed by visual inspection.

**Empirical heuristic performance for 304 search queries with all candidate concepts *Y_Tx_* with evidence.**

- With a threshold n4 >7, the recall is 100%. All search queries with n4 >7 (the best *clinical winners*) are selected by the heuristic. The precision was 4.30% (the lowest value).
- With a threshold n4 >0 (at least one *Y_Tx_* with therapeutic intent), the precision was 97.85% (the highest value) and the recall was 32.50%.
- With a threshold n4 >2, where 3 was the lowest value among the *clinical winners* (Tables 3 and 4), the precision was 78.49% and the recall was 39.67%.

### Limitations

Our work relies on semantic fields and has 2 main limitations [62]: (1) there are overlaps of meaning and (2) there are gaps in meaning. This has 2 clear implications for the lists of concepts *Y_Tx_* per disease x:

- The lists may not comprise mutually exclusive concepts in meaning. For example, “C0060657|formoterol” and “C1276807|Budesonide/formoterol” are both treatments with evidence of therapeutic intent for asthma [63].
- The lists were incomplete. For example, “C0772501|Levalbuterol” and “C0907850|ciclesonide” are both treatments with evidence of therapeutic intent for asthma [63] and not among the *Y_Tx_* for asthma.

We did not use Skip-gram with negative sampling (also known as SGNS); therefore, it can be argued that we did not use the best configuration of a word2vec model [21]. The effect of hyperparameter configurations appeared in studies by Levy et al and Chiu et al [64,65], and Allen and Hospedales [66] reviewed mathematical proofs and equations with an emphasis on SGNS for pair-based proportional analogies.

For Stage 1, the 3CosAdd formula needed at least two n-gram pairs (disease *x*, treatment *z*) [29]. Only one search query could be made for the target disease, chronic kidney disease and diabetes, and none for obesity. Other studies that replicated the application to the 3CosAdd formula for target disease *x* could suffer the same limitation. For example, in Appendix B in the study by Pakhomov et al [67], among the 100 top-ranked candidate terms (highest cosine value) “semantically similar or related” to target disease “heart failure”, there were no treatments (ie, Tx encompassing 3 textual definitions from Hart et al [24]).

For Stage 2, the MetaMap version was 2016v2 (with a 2016 UMLS release), and few n-grams were considered as clear terminological gaps. The n-gram “anti-VEGF_agents” was manually mapped to CUI=C4727875, which exists in the 2019AA UMLS release. Five n-grams were mapped to very broad CUIs as they had the character “*” in Multimedia Appendix 2 (worksheet Stage 3).

The NER task (Stage 2) and the searchers in the literature seeking evidence for concept pairs (Stage 3) were time-consuming and required highly trained domain experts. The appraisal of the literature was not performed by a review team as proficient as the ones conducting Cochrane systematic reviews.

The heuristic developed by visual inspection lacked finesse, and its improvement calls for further investigation.

### Comparison With Prior Work

The UMLS CUIs were mapped to SNOMED CT identifiers [30]. From a “digital health care” perspective [68], the UK NHS is moving toward the adoption of SNOMED CT as the only terminology for all care settings [69]. A subset of SNOMED CT concepts under worldwide adoption is the CORE Problem List Subset of SNOMED CT [70], and the UK NHS has developed 2 human-readable SNOMED CT subsets [71]: UK Clinical Extension and UK Drug Extension. However, SNOMED CT lacks statements representing the treatments that can be considered for a disease (eg, inhaled corticosteroid treats asthma) and, to the best of the authors’ knowledge, there are no SNOMED CT subsets for well-known diseases.

There are reusable datasets for evaluating relatedness made of UMLS CUI pairs:

- Medical coders set [72]: 101 CUI pairs mapped to terms, typically multiple words. Only 29 pairs have a high interrater agreement.
- Medical Residents Relatedness Set [73]: 588 CUI pairs mapped to terms, typically single words. Using single words is a severe limitation as “most medical terms consist of more than one word” [67].
- UMLS MRREL table [58]: It has relationships asserted by source vocabularies between CUI pairs. Among the relationship attributes appear the following: “may_prevent”, “may_treat”, and “has_contraindicated_drug”.

All reusable datasets mentioned above lack evidence (quotes with references) from the biomedical literature. Multimedia Appendix 1 cross-compares these reusable datasets and the 408 UMLS CUI pairs investigated thoroughly in this study.

### Conclusions

Extracting clinically useful information automatically from free text in PubMed/MEDLINE may require a natural language understanding of statements containing relevant relations for health care. Hence, extracting treatments with therapeutic intent by analogical reasoning from embeddings (423K n-grams from the PMSB dataset) is an ambitious goal. Our SemDeep approach is knowledge-based, underpinned by embedding analogies that exploit prior knowledge. Biomedical facts from embedding analogies (a 4-term type, not pairwise) are potentially useful for clinicians. The heuristic offers a practical way to discover beneficial treatments for well-known diseases.

Learning from deep learning models does not require a massive amount of data. Embedding analogies are not limited to pairwise analogies; hence, analogical reasoning with embeddings is underexploited.

## Appendix

Multimedia Appendix 1 Additional material.

Multimedia Appendix 2 Data to replicate the results reported.

## Abbreviations

BMJ: British Medical Journal
CBOW: Continuous Bag-of-Words
CUI: concept unique identifier
MRREL: the UMLS related concepts table (file=MRREL)
NER: named entity recognition
NHS: National Health Service
PMSB: PubMed systematic reviews subset
SemDeep: Semantic Deep Learning
SGNS: skip-gram with negative sampling
SNOMED CT: systematized nomenclature of medicine - clinical terms
Tx: treatment
UMLS: unified medical language system
URIs: Universal Resource Identifiers
