# Supplementary material for: Semantic Deep Learning: Prior Knowledge and a Type of Four-Term Embedding Analogy to Acquire Treatments for Well-Known Diseases
Source: JMIR Med Inform. 2020 Aug 6;8(8):e16948. doi: 10.2196/16948 (PMC7441383; doi:10.2196/16948)
Supplement: Multimedia Appendix 1 [file medinform_v8i8e16948_app1.pdf]

Multimedia Appendix 1

Index of additional material

1. Analogies for Shakespeare's Romeo in a small common-English corpus

1.1 The baseline corpus and pre-processing … page 2

1.2 Creating "good" embeddings: answering Q1 … page 2

1.3 Finding analogies using Romeo as a target: answering Q2 … … … … … … … … … … … … … … page 3

2. The three stages of our SemDeep approach

2.1 Stage 1 Knowledge acquisition (acquisition of domain-specific terms)

2.1.1 Prior knowledge … page 3-4

2.2. Stage 2 Knowledge organisation (explicit conceptualisation of the meaning of terms)

2.2.1 Methods: the three sequential subtasks under NER … … … … … … … … … … … … … … page 5

2.2.2 Results: the three sequential subtasks under NER … … … … … … … … … … … … … … … page 5-7

2.2.3 Investigating the UMLS Semantic Types for the UMLS Metathesaurus concepts $\mathbf{Y_{Tx}}$ … … page 7-8

2.3 Stage 3 Knowledge validation (validating statements)

2.3.1 Evaluation guidelines given to observers for Stage 3 … … … … … … … … … … … … … page 9

2.3.2 Further details of the evidence-based validation of UMLS Metathesaurus concept pairs … page 10-12

2.3.3 Cohen's Kappa (including paradox resolution) … … … … … … … … … … … … … … … … … page 12

2.3.4 Illustrating a best clinical winner that is also a NER winner … … … … … … … … … … … … page 13

2.3.5 Source data for Figure 3 … page 13

3. Cross-comparison with reusable datasets for evaluating relatedness… … …page 14-15

References … page 15-16

1. Analogies for Shakespeare's Romeo in a small common-English corpus

1.1 The baseline corpus and pre-processing

We take as a baseline corpus the one introduced by A. Thomo in a tutorial for a topic model [1]. The pre-processing to be performed over the corpus (e.g. removing stop words) can affect the candidate words yielding the highest value for both the cosine and the 3CosAdd formula [2,3].

Textbox 1 contains the corpus as input for the word2vec software [4], and it can be downloaded as a text file from [5]. The corpus from [1] consists of 5 documents, each document is a line of text. As can be observed in Textbox 1:

- The text is not lower-case and stop words (e.g. subject pronouns like you) are not removed.
- Most of the punctuation marks have not been removed. A white space has been added before the punctuation marks as well as the beginning and end of each sentence (line of text).
- Quotation marks (e.g. "") have been removed, although the contractions (e.g. 's) have not.

Textbox 1 – The common-English small corpus after the pre-processing

```
Romeo and Juliet
Juliet : Oh happy dagger !
Romeo died by dagger .
Live free or die , that ' s the New-Hampshire ' s motto
Did you know New-Hampshire is in New-England ?
```

1.2 Creating "good" embeddings: answering Q1

We interpret as a "good" embeddings those performing well in semantic similarity and relatedness tasks. A "good" vector semantic model should be able to find a candidate word **y** that is a "semantic similar" to the target word **x** = **Romeo**. We use cosine (1) as the similarity measure.

$$\cos(\mathbf{x},\mathbf{y}) = (\mathbf{x} \cdot \mathbf{y}) / (||\mathbf{x}|| \, ||\mathbf{y}||) \qquad (1) \text{ [2]}$$

Textbox 2 contains a command line, invoking the executable code for the file word2vec, with the values for the variables (hyperparameters) of the vector semantics model. As can be seen from Textbox 2, four pre-processing hyperparameters need to be considered:

- Vector dimension is 5, i.e. "–size 5".
- Context window size is 3, i.e. "-window 3".
- Subsampling is left with the default value, "-sample 1e-5".
- Minimum count is 1, i.e. "-min-count 1". Hence, no word will be discarded.

Textbox 2 – The command line to create the CBOW model

```
./word2vec -train Romeo_inputDocs.txt -output Romeo_emb.bin -save-vocab Romeo_voc.txt -debug 2 -size 5 -
window 3 -min-count 1 -sample 1e-5 -hs 0 -binary 1 -cbow 1
```

The two association metric hyperparameters are left at the default values:

- Negative sampling ("-negative") is not used.
- Learning rate ("-alpha") is left at the default value of 0.025.

The word2vec model is CBOW (Continuous Bag-of-Words) [3] ("-cbow 1"). The vectors created are saved in the binary file "Romeo_emb.bin". We use the target word **x** = **Romeo** to obtain the candidate word **y** with the highest cosine value:

Word	Cosine
you	0.644935

The word **you** is a personal pronoun, and the word **Romeo** is a proper noun. The words **you** and **Romeo** can be interpreted as near-synonyms as they can be used "interchangeably in some contexts" [6]. The word **you** can be a subject pronoun as well as an object pronoun. Examples: "You (subject) are Romeo. I saw you (object) yesterday. The person I love is you (object), Romeo". Hence, the model created with CBOW is interpreted as "good" embeddings, i.e. it performs well in the semantic similarity and relatedness tasks.

1.3 Finding analogies using Romeo as a target: answering Q2

We utilise the 3CosAdd formula as in [2], although interpreting the formula as "find the word **y**, which is similar to the word **z1** and the word **z2**, while different from the word **x**", where **x** provides the semantic context. The 3CosAdd formula is rewritten below (2) as:

$$\arg\max(\cos(\mathbf{y},\mathbf{z1}) - \cos(\mathbf{y},\mathbf{x}) + \cos(\mathbf{y},\mathbf{z2})) \qquad (2)$$

We apply the 3CosAdd formula, where **Romeo** = **x** provides the semantic context. The first 3 documents (lines of text) in the corpus seem taken from Romeo and Juliet, a famous theatre play by Shakespeare. The words **die** = **z1** and **died** = **z2** appear in the corpus and are representative of inflectional morphology **infinitive:past**. Hence, the query we pose to the CBOW model is "find the word **y**, which is similar to **die** and **died**, while different from **Romeo**". The candidate word **y** with the highest value for the 3CosAdd formula:

Word	3CosAdd
dagger	0.858598

It is easy to envision the creation of a semantic field death containing the words {die; died; dagger}, i.e. a set of words that "belong together under the same conceptual heading" [7]. The noun "death" and the verb "die" are in the same lexical field, but are different parts of speech. The word pairs **die:dagger** or **died:dagger** are closely related (dagger is an instrument that causes death).

In conclusion, a vector semantics model created with CBOW can find analogies in a very small corpus.

2. The three stages of our SemDeep approach

2.1 Stage 1 Knowledge acquisition (acquisition of domain-specific terms)

2.1.1 Prior knowledge

The stage 1 utilises prior knowledge (open-access reusable datasets from [8]), consisting of n-gram pairs obtained by applying the cosine to embeddings, then mapped to the Unified Medical Language System (UMLS) Metathesaurus [9] concept pairs, and finally validated with evidence from biomedical literature using BMJ Best Practice [10] as the main information source.

BMJ Best Practice is outside PubMed/MEDLINE [11], and is acknowledged for its editorial quality and for evidence-based methodology [12]. In the UK, BMJ Best Practice is provided (free access) to all NHS healthcare professionals in England, Scotland, and Wales [13]. BMJ Best Practice provides advice on symptom evaluation, tests to order and treatment approach structured around the patient consultation. The advice is organised into condition and assessment topics and is synthesized manually from clinical practice guidelines, systematic reviews and clinical trials. It uses expert contributors and editors, and requires a subscription.

Table 1 – UMLS CUIs and UMLS Semantic Types for target n-grams **x**, mapped to SNOMED CT identifiers

Disease Target **x**	Target concept **X** UMLS CUI	Target concept **X** SNOMED CT identifier	UMLS Semantic Type for UMLS CUI of target concept **X**
heart_failure	C0018801	84114007	T047
glaucoma	C0017601	23986001	T047
CKD	C1561643	709044004	T047
diabetes	C0011849	73211009	T047
asthma	C0004096	195967001	T047
epilepsy	C0014544	84757009	T047
arthritis	C0003864	3723001	T047
osteoarthritis	C0029408	396275006	T047
anaemia	C0002871	271737000	T047
hypertension	C0020538	38341003	T047

Table 1 contains the target n-gram **x** belonging to the semantic field disease. Each target n-gram **x** represents a medical diagnosis mappable to a "type-of" of SNOMED CT [14] concept called disorder. Table 1 contains the UMLS CUI as well as the SNOMED CT identifier for each n-gram **x**. The UMLS Semantic Type for the UMLS Metathesaurus concept **X** mapped to target disease n-gram **x** is "T047|Disease or Syndrome".

Table 2 – Prior knowledge: the 36 unique (disease **X**,treatment **Z**) UMLS CUI pairs. The last column shows the UMLS Semantic Types for the UMLS Metathesaurus concept **Z**

Disease Target **x**	Target concept **X** UMLS CUI	Treatment concept **Z** UMLS CUI	UMLS Semantic Type for UMLS CUI of concept **Z**
heart_failure	C0018801	C0001645	T121
heart_failure	C0018801	C0002007	T121
heart_failure	C0018801	C0003015	T121
heart_failure	C0018801	C1167956	T061
glaucoma	C0017601	C0007389	T061
glaucoma	C0017601	C0040574	T061
CKD	C1561643	C0011946	T061
diabetes	C0011849	C0017725	T109,T123,T121
diabetes	C0011849	C2917359	T121
asthma	C0004096	C0595726	T109,T121
asthma	C0004096	C2936789	T121
asthma	C0004096	C4020618	T121
epilepsy	C0014544	C0003299	T121
epilepsy	C0014544	C0080356	T109,T121
epilepsy	C0014544	C0377265	T109,T121
arthritis	C0003864	C0025677	T109,T121
arthritis	C0003864	C0242708	T121
arthritis	C0003864	C1257954	T109,T121
arthritis	C0003864	C2064783	T061
osteoarthritis	C0029408	C0000970	T109,T121
osteoarthritis	C0029408	C0017718	T109,T121
osteoarthritis	C0029408	C0020196	T109,T123,T121
osteoarthritis	C0029408	C0021488	T061
osteoarthritis	C0029408	C0086511	T061
osteoarthritis	C0029408	C2350507	T061
anaemia	C0002871	C0005841	T061
anaemia	C0002871	C0014822	T116,T125,T121
anaemia	C0002871	C0302583	T123,T121,T196
anaemia	C0002871	C0376541	T116,T121
anaemia	C0002871	C0937950	T116,T121
anaemia	C0002871	C1959590	T121
hypertension	C0020538	C0003364	T121
hypertension	C0020538	C0006684	T121
hypertension	C0020538	C0521942	T121
hypertension	C0020538	C0585941	T061
hypertension	C0020538	C2960841	T061

Table 2 shows the 36 unique UMLS CUI pairs (disease **X**,treatment **Z**) taken from additional file 3 in [8]. The treatment CUIs **Z** are mapped to n-grams **z** belonging to the semantic field treatment (Tx for short). The n-gram **z** reused by the current study (i.e. z1 and z2) are from additional file 1 in [8]. Every reused n-gram **z** is mapped to UMLS concept **Z** with the UMLS Semantic Type "T061|Therapeutic or Preventive Procedure" or "T121|Pharmacologic Substance".

2.2 Stage 2 Knowledge organisation (explicit conceptualisation of the meaning of terms)

2.2.1 Methods: the three sequential subtasks under NER

The stage 2 accomplishes a task known as Named Entity Recognition (NER) [15], comprising three sequential subtasks (detailed below), and involving three domain experts: two biomedical terminologists (rater A and B) and a medical consultant with many years of experience doing clinical coding (regarded as an empirical gold standard or rater GS for short).

Firstly, disambiguation of n-grams **y** difficult to interpret for being truncated strings of characters or containing short forms (e.g. abbreviations or acronyms). Using the disease **x** as context, more information for candidate treatment **y** is obtained from manual searches: string of characters exact-match searches in the PMSB dataset; and/or Web searches the sense inventory Allie [16] looking for short-long form pairs. For example, for the unigram "HES" (i.e. a short form), a Web search with Allie brings as the top-1 result the long form "hydroxyethyl starch".

Secondly, manual binary classification of candidate n-gram **y** as to whether or not it belongs to the semantic field Tx (i.e. $\mathbf{y}_{\mathbf{Tx}}$). Section 2.3.1 (page 9) reproduces the three textual definitions from [17] encompassing Tx along with examples. We report the interrater agreement between raters following [18]: calculating Cohen's Kappa measure [19] for each pair of raters, and Krippendorff's alpha [20] (a generalised version) for more than two raters.

Thirdly, entity normalisation (a.k.a. grounding) [21] with MetaMap [22] where three domain experts follow the NER guidelines for MetaMap's output from [23] and together judge the automatic mapping of n-grams $\mathbf{y}_{\mathbf{Tx}}$ to UMLS Metathesaurus concepts $\mathbf{Y}_{\mathbf{Tx}}$. For each n-gram, MetaMap provides: a) a single CUI; b) multiple CUIs (either a single list of CUIs or multiple lists of CUIs); and c) no CUI. MetaMap produces two types of errors: missing (False Negative or FN) and spurious (False Positive or FP). MetaMap performance is assessed with the conventional evaluation measures [24]: Precision = TP/(TP + FP), Recall = TP/(TP + FN), and F measure = (2 x Precision x Recall)/(Recall + Precision). As in [25], we calculate a weaker precision and recall where an exact match and a partial match are equally counted as a TP. The calculations ignore the number of CUIs provided by MetaMap for an n-gram. Precision, recall, and F measure are calculated per target disease **x**. We also report macro-averaging (by averaging each evaluation measure) [24] and micro-averaging (by making a single contingency table for all data) [24].

2.2.2 Results: the three sequential subtasks under NER

Disambiguation of n-grams

Column C in Multimedia Appendix 2 (worksheet Stage2) summarises the manual searches performed for the 467 n-gram pairs with a candidate **y** difficult to interpret. Multimedia Appendix 2 (worksheet "Disambiguation n-grams") has the expanded strings and further information for truncated strings (71 candidates n-gram **y**).

Multimedia Appendix 2 (worksheet "Disambiguation SF to LF") contains the Long Form (LF) for each short form (SF) of 253 abbreviations or acronyms appearing within the candidates n-gram **y**. Allie [16] performance to obtain a SF for a LF is calculated taking as TP a top-1 result that is a correct long form. The F measure is 87.31% with precision =84.85% and recall 89.91%.

Binary classification (Tx or non Tx)

Among the 1935 unique (**x**,**y**) n-gram pairs, there are 954 n-gram pairs (**x**, $\mathbf{y}_{\mathbf{Tx}}$) with candidate **y** belonging to Tx. Table 3 shows the number of unique candidates **y** and $\mathbf{y}_{\mathbf{Tx}}$ per model and disease target **x**. Table 4 contains the results of the binary classification (Tx as 1 and non Tx as 0) of candidates **y** per disease target **x** and the interrater agreement with Cohen's Kappa [19] and Krippendorff's alpha [20]. The three domain experts perform the binary classification independently for six of the ten diseases, i.e. for 627 candidate **y**. For the remaining four diseases, i.e. for 1308 candidate **y**, the three raters work collaboratively in the manual binary classification of n-grams.

Table 3 – Binary classification (Tx as 1 and non Tx as 0) of candidate **y** and $\mathbf{y_{Tx}}$ per model and disease target **x**

Disease Target **x**	Number search queries	Number (**x**,**y**) pairs	CBOW Number Candidate **y**	Skip-gram Number Candidate **y**	CBOW Number Candidate $\mathbf{y_{Tx}}$	Skip-gram Number Candidate $\mathbf{y_{Tx}}$
heart_failure	10	120	65	51	44	38
glaucoma	3	36	30	28	14	17
CKD	1	12	12	12	9	8
diabetes	1	12	12	12	10	10
asthma	36	432	144	79	74	70
epilepsy	36	432	200	85	84	49
arthritis	10	120	89	70	39	38
osteoarthritis	15	180	126	73	62	51
anaemia	66	792	407	206	149	140
hypertension	45	540	301	152	112	98

Table 4 – Binary classification (Tx as 1 and non Tx as 0) of unique candidates **y** per disease target **x**. The last columns show the interrater agreement (IAA) considering columns D, E, and F within Multimedia Appendix 2 (worksheet Stage2).
Abbreviations: GS = rater GS (medical consultant); A = rater A (terminologist); B = rater B (terminologist)

Disease Target **x**	Number Candidate **y**	Number Candidate **y** as Tx ($\mathbf{y_{Tx}}$)	Number Candidate **y** as non Tx	Cohen's Kappa IAA(GS,A)	Cohen's Kappa IAA(GS,B)	Cohen's Kappa IAA(A,B)	Krippendorff's alpha IAA(GS,A,B)
heart_failure	101	69	32	0.55	0.89	0.58	0.67
Glaucoma	47	25	22	0.92	0.83	0.75	0.83
CKD	21	15	6	1.00	0.89	0.89	0.93
diabetes	20	17	3	1.00	0.58	0.58	0.69
asthma	192	115	77	0.91	0.90	0.90	0.91
epilepsy	246	109	137	0.88	0.94	0.88	0.90
arthritis	146	67	79				
osteoarthritis	179	99	80				
anaemia	556	250	306				
hypertension	427	188	239				

Considering all candidates **y** for six of the ten diseases, i.e. total of 627 candidates **y**, Krippendorff's alpha [20] is 0.86 for the three raters. The Cohen's Kappa [19] for each pair of raters: IAA(GS,A) = 0.86; IAA(GS,B) = 0.91; IAA(A,B) = 0.83. The three Cohen's Kappa values are interpreted as "almost perfect agreement" [26].

Normalisation with MetaMap

The 954 n-gram pairs (**x**, $\mathbf{y_{Tx}}$) with candidate **y** belonging to Tx, were mapped to 569 unique ($\mathbf{X}$,$\mathbf{Y_{Tx}}$) UMLS CUI pairs. Table 5 shows the MetaMap performance per disease target **x** in mapping candidates $\mathbf{y_{Tx}}$ to UMLS Metathesaurus concepts $\mathbf{Y_{Tx}}$. The overall measure of performance (macro-averaging) [24] of MetaMap by averaging each evaluation measure from Table 5 over all ten diseases: F measure = 78.50% with precision = 77.69% and recall = 82.46%. The candidates **y** for the target disease **x** = heart_failure are particularly challenging, with the lowest interrater agreement for the binary classification (Table 4) and the lowest F measure for MetaMap (Table 5).

Considering all candidate $\mathbf{y_{Tx}}$ for the ten diseases, the micro-averaging performance (making a single contingency table for all data) [24] of MetaMap is: F measure=80.00% with precision=77.00% and recall =83.25%. If all candidate $\mathbf{y_{Tx}}$ that are truncated strings or contained short forms are not considered, the MetaMap micro-averaging performance increases by 10% to F measure=90.88%, with precision=90.73% and recall=91.03%.

Table 5 – MetaMap performance per disease target **x** when mapping candidates $\mathbf{y}_{Tx}$ to UMLS Metathesaurus concepts $\mathbf{Y}_{Tx}$ using the values in column H within Multimedia Appendix 2 (worksheet Stage2)

Disease Target **x**	Number Candidate $\mathbf{y}_{Tx}$	TP	FP	FN	Precision %	Recall %	F measure %
heart_failure	69	30	4	35	88.24	46.15	60.61
glaucoma	25	22	2	1	91.67	95.65	93.62
CKD	15	9	2	4	81.82	69.23	75.00
diabetes	17	9	6	2	60.00	81.82	69.23
asthma	115	58	50	7	53.70	89.23	67.05
epilepsy	109	87	17	5	83.65	94.57	88.78
arthritis	67	54	9	4	85.71	93.10	89.26
osteoarthritis	99	65	28	6	69.89	91.55	79.27
anaemia	250	182	47	21	79.48	89.66	84.26
hypertension	188	120	25	43	82.76	73.62	77.92

2.2.3 Investigating the UMLS Semantic Types for the UMLS Metathesaurus concepts $\mathbf{Y}_{Tx}$

The 954 n-gram pairs (**x**, $\mathbf{y}_{Tx}$) with candidate **y** belonging to Tx, were mapped to 569 unique ($\mathbf{X}$,$\mathbf{Y}_{Tx}$) UMLS CUI pairs. This study considers the UMLS Semantic Types (133 broad categories) [27] to classify UMLS Metathesaurus concepts $\mathbf{Y}_{Tx}$. The rationale for an investigation of the UMLS Semantic Types for the UMLS Metathesaurus concepts $\mathbf{Y}_{Tx}$ is that the set of the Semantic Types assigned to them may allow a machine-processable definition for Tx instead of the three textual human-readable definitions from [17].

We investigate a further categorisation of concepts $\mathbf{Y}_{Tx}$ based on the UMLS Semantic Types assigned. Table 6 shows how these 6 main categories (first column) can be further decomposed into 11 subcategories (second column) considering 38 UMLS Semantic Types (last column) of the total 133 UMLS Semantic Types [27]. The 11 subcategories from Table 6 appear in column I of Multimedia Appendix 2 (worksheet Stage2).

Table 6 – Six main categories introduced (last six columns of Table 7 and 8), their further decomposition into subcategories, and their mapping to UMLS Semantic Types

Main category based on UMLS Semantic Types	Subcategory based on UMLS Semantic Types	UMLS Semantic Type
T061 or T121	T061	T061
T061 or T121	T121	T121
T058	T058	T058
T070	T070	T070
Device	Device (T074)	T074
Device	Device (T073)	T073
Nutrition and Lifestyle	Nutrition and Lifestyle	T168,T007,T002,T056,T054
Substance	Substance (T195)	T195
Substance	Substance (T167)	T167
Substance	Substance (T031)	T031
Substance	Substance (CHEM)	T116,T123,T122,T118,T103,T120,T104,T200, T111,T196,T126,T131,T125,T129,T130,T197, T119,T124,T114,T109,T115,T192,T110,T127

Table 6 shows how the six main categories produced consisting of one Semantic Type, i.e. "T058|Health Care Activity" and "T070|Natural Phenomenon or Process", or more than one Semantic Type, e.g. "T061 or T121" or "Device". The subcategory "Substance (CHEM)" takes most of the UMLS Semantic Types from the UMLS Semantic Group "Chemicals & Drugs" [27].

Table 7 – Categorisation of UMLS CUIs for candidate concepts $\mathbf{Y_{Tx}}$ (third column) into the 6 main categories introduced (last six columns) based on 38 UMLS Semantic Types. The second column shows the number of candidate n-grams $\mathbf{y_{Tx}}$ mapped to those UMLS CUIs $\mathbf{Y_{Tx}}$ (third column).

			Categorisation of UMLS CUIs for candidate concepts $\mathbf{Y_{Tx}}$					
Disease Target **x**	Number Candidate $\mathbf{y_{Tx}}$	Number Candidate concept $\mathbf{Y_{Tx}}$ (UMLS CUI)	T061 or T121	T058	T070	Device	Substance	Nutrition and Lifestyle
heart_failure	69	41	24	1	1	2	13	0
glaucoma	25	19	12	2	1	0	4	0
CKD	15	10	9	0	0	0	1	0
diabetes	17	12	4	0	0	0	8	0
asthma	115	49	23	1	0	1	23	1
epilepsy	109	76	35	0	0	1	40	0
arthritis	67	37	14	2	0	2	18	1
osteoarthritis	99	71	38	0	0	0	30	3
anaemia	250	144	49	4	0	4	79	8
hypertension	188	110	47	5	0	6	48	4
Total	954	569	255	15	2	16	264	17

Table 8 – Categorisation of candidate n-grams $\mathbf{y_{Tx}}$ considering their mapping to UMLS CUIs for candidate concepts $\mathbf{Y_{Tx}}$ and the UMLS Semantic Types assigned to those UMLS CUIs

		Categorisation of candidate n-grams $\mathbf{y_{Tx}}$					
Disease Target **x**	Number Candidate $\mathbf{y_{Tx}}$	T061 or T121	T058	T070	Device	Substance	Nutrition and Lifestyle
heart_failure	69	53	1	1	2	12	0
Glaucoma	25	18	2	1	0	4	0
CKD	15	13	0	0	0	2	0
diabetes	17	9	0	0	0	8	0
asthma	115	79	1	0	4	30	1
epilepsy	109	59	0	0	1	49	0
arthritis	67	29	2	0	2	33	1
osteoarthritis	99	56	0	0	0	41	2
anaemia	250	104	6	0	4	128	8
hypertension	188	110	5	0	6	62	5
Total	954	530	17	2	19	369	17

From Table 7 we can observe that the two main categories with the higher number of UMLS CUIs are: "Substance" with 264 candidate concepts $\mathbf{Y_{Tx}}$ and "T061 or T121" with 255 candidate concepts $\mathbf{Y_{Tx}}$. However, if we take into account Table 8 and the n-grams $\mathbf{y_{Tx}}$ mapped to UMLS CUIs $\mathbf{Y_{Tx}}$, we can observe that the higher number of candidate n-grams $\mathbf{y_{Tx}}$ is for the main category "T061 or T121" with 530 of the total 954.

From our investigation of the UMLS Semantic Types assigned to the candidate concepts $\mathbf{Y_{Tx}}$, mapped to the candidate n-grams $\mathbf{y_{Tx}}$, we conclude that the higher number of n-grams $\mathbf{y_{Tx}}$ with candidate **y** belonging to Tx, is for the main category "T061 or T121", i.e. the main category that has the two UMLS Semantic Types considered for the n-grams **z** used as prior knowledge.

2.3 Stage 3 Knowledge validation (validating statements)

2.3.1 Evaluation guidelines given to observers for Stage 3

Task: Express agreement or disagreement for the categories (evidence-based labels) assigned to biomedical concepts related to medical conditions

Biomedical/clinical definitions
We consider the following definitions from [17]:

- **Treatment** – Action taken by a health professional, in the context of contact with a treatment recipient, to alter the functioning of an individual with a disability or at risk of a disability. Treatment is defined broadly to include provision of information, devices, and referrals, specific active experiences, and passive interventions.
- **Treatment ingredients** – Observable (and, therefore, in principle, measurable) actions, chemicals, devices, or forms of energy that are selected or delivered by the clinician.
- **Mechanism of action** – Process by which the treatment's essential ingredients induce change in the target of treatment.

Under "mechanism of action" of a treatment, we consider the active ingredients as well as the body's cells or substances and their interplay. Let's consider a detailed example:

- T helper cells (a.k.a. T4 cells because they have the CD4 surface molecule) stimulate the production of antibodies against pathogens, and thus, the loss or improper function of CD4 T cell responses is detrimental for immunity to a wide range of pathogens [28].
- "The understanding of how sepsis affects CD4 T cells through their numerical loss and recovery, as well as function, is important in the development of future treatments designed to restore CD4 T cells to their presepsis state." [28].
- In this study, CD4 T cells is interpreted as a "treatment ingredient" as CD4 T cells play a role in the "mechanism of action" of treatments designed to restore CD4 T cells.

Other definitions: correlation
In this study we use the term "correlation" taking into account the definition for "is correlated with" from [29]:
"*x is correlated with y when x holds a statistical relationship with y*"

Let's exemplify its use:
"*The correlation between genotype (from the Greek genos, meaning race, offspring) and phenotype (from the Greek phaino-, from phainein, meaning to show) is defined as an above-chance probability of a distinct mutation being associated with a particular physical feature or abnormality. The genotype and phenotype share a statistical relationship."* [30]

The above-mentioned correlation will be represented as: Correlation (**genotype → phenotype**)

Note that more than one correlation can be represented, such as Correlation (**A → B → C**), where the arrow (i.e. →) indicates the direction of reading

Plausible reasons for disagreement
Among others, we acknowledge the following reasons to express disagreement:

- The evidence (quoted text) provided is somewhat unclear.
- The domain knowledge and clinical expertise of the biomedical/clinical expert based on clinical practice
- The domain knowledge and clinical expertise of the biomedical/clinical expert based on evidence-based literature (e.g. a more recent paper)

Note: *A simplified version (excluding UMLS CUIs) of the table displayed is included in the main manuscript as Table 1, exemplifying the seven evidence-based categories introduced.*

2.3.2 Further details of the evidence-based validation of UMLS Metathesaurus concept pairs

Only 68 of the 569 UMLS CUI pairs are in the MRREL table [31] of UMLS 2019AA, see column J within Multimedia Appendix 2 (worksheet Stage3). Table 9 shows the CUI pairs found in MRREL table per disease target **x**.

Table 9 – Number of UMLS CUI pairs within the MRREL table [31] of UMLS 2019AA

Disease Target **x**	Target concept **X** UMLS CUI	Number Candidate concept $\mathbf{Y}_{\mathbf{Tx}}$ (UMLS CUI)	Number of UMLS CUI pairs ($\mathbf{X}$, $\mathbf{Y}_{\mathbf{Tx}}$) or ($\mathbf{Y}_{\mathbf{Tx}}$, $\mathbf{X}$) in MRREL table of UMLS 2019AA
heart_failure	C0018801	41	7
glaucoma	C0017601	19	3
CKD	C1561643	10	1
diabetes	C0011849	12	0
asthma	C0004096	49	9
epilepsy	C0014544	76	12
arthritis	C0003864	37	0
osteoarthritis	C0029408	71	9
anaemia	C0002871	144	13
hypertension	C0020538	110	14
Total	10	569	68

Table 10 shows the total number of search queries per disease target **x**. Only 304 search queries (144 for CBOW and 160 for Skip-gram) of the total 446 (223 for CBOW and 223 for Skip-gram) have all the candidate $\mathbf{y}_{\mathbf{Tx}}$ mapped to concepts $\mathbf{Y}_{\mathbf{Tx}}$ with at least one evidence-based category assigned.

Table 10 displays the number of total candidate concepts $\mathbf{Y}_{\mathbf{Tx}}$ per disease target **x** (column previous to last), and the number of those with evidence (last column). By cross-comparing the last two columns of Table 10, it can be observed that there are some UMLS concept pairs ($\mathbf{X}$,$\mathbf{Y}_{\mathbf{Tx}}$) for anaemina and hypertension not investigated thoroughly. Indeed, only 37 CUI pairs for **x**=anaemina and 56 CUI pairs for **x**=hypertension are investigated thoroughly.

Table 10 – Number of search queries per disease target **x**, and number of concept pairs ($\mathbf{X}$, $\mathbf{Y}_{\mathbf{Tx}}$) with evidence

Disease Target **x**	Total Number search queries	CBOW Number search queries with evidence for all Candidate concept $\mathbf{Y}_{\mathbf{Tx}}$	Skip-gram Number search queries with evidence for all Candidate concept $\mathbf{Y}_{\mathbf{Tx}}$	Number Candidate concept $\mathbf{Y}_{\mathbf{Tx}}$ (UMLS CUI)	Number Candidate concept $\mathbf{Y}_{\mathbf{Tx}}$ (UMLS CUI) with evidence
heart_failure	10	10	10	41	41
glaucoma	3	3	3	19	19
CKD	1	1	1	10	10
diabetes	1	1	1	12	12
asthma	36	36	36	49	49
epilepsy	36	36	36	76	76
arthritis	10	10	10	37	37
osteoarthritis	15	15	15	71	71
anaemia	66	10	24	144	37
hypertension	45	22	24	110	56
Total	223	144	160	569	408

Table 11 shows the 408 concept pairs ($\mathbf{X}$,$\mathbf{Y}_{\mathbf{Tx}}$) investigated thoroughly per target disease **x** according to the seven evidence-based categories proposed in this study. Multimedia Appendix 2 (worksheet Stage3) contains the evidence (quoted text in column H) and their references (column I). The highest number of concept pairs ($\mathbf{X}$,$\mathbf{Y}_{\mathbf{Tx}}$) with evidence provided is for the category "Tx with therapeutic effect" with 190 concept pairs, followed by the evidence-based category "Correlation" with 94 concept pairs.

If we look at Table 11 focusing per target disease **x**, the number of concept pairs (**X**,$\mathbf{Y}_{\mathbf{Tx}}$) for the evidence-based category "Tx with therapeutic effect" is always systematically higher than any other evidence-based category except for target disease **x**=arthritis, where the category "Tx with therapeutic effect" has the same number of concept pairs as the evidence-based category "Tx with uncertain therapeutic effect".

Table 11 – Number of UMLS Metathesaurus concept pairs (**X**, $\mathbf{Y}_{\mathbf{Tx}}$) per evidence-based category

		Number of UMLS Metathesaurus concept pairs (**X**, $\mathbf{Y}_{\mathbf{Tx}}$)						
Disease Target **x**	Number Candidate concept $\mathbf{Y}_{\mathbf{Tx}}$ (UMLS CUI) with evidence	Tx with therapeutic effect	Tx with uncertain therapeutic effect	Tx with unwanted or adverse effects	Potential Tx	Tx ingredient	General medical term	Correlation
heart_failure	41	17	2	7	0	5	5	7
Glaucoma	19	12	0	1	0	0	2	5
CKD	10	8	0	0	0	0	1	1
diabetes	12	10	0	1	0	2	0	0
asthma	49	24	7	5	1	3	2	8
epilepsy	76	23	3	22	2	5	6	18
arthritis	37	10	10	3	0	1	5	8
osteoarthritis	71	39	12	3	0	5	2	16
anaemia	37	19	1	1	0	0	0	16
hypertension	56	28	3	9	2	1	3	15
Total	408	190	38	52	5	22	26	94

There are 19 concept pairs (**X**,$\mathbf{Y}_{\mathbf{Tx}}$) of the 408 concept pairs (**X**,$\mathbf{Y}_{\mathbf{Tx}}$) investigated thoroughly with more than one evidence-based category assigned, like the concept pair (**X**=C0014544|Epilepsy,$\mathbf{Y}_{\mathbf{Tx}}$=C0080356|Valproate).

Table 12 – The 408 UMLS CUI pairs investigated thoroughly and their evidence-based information sources per evidence-based category

Evidence-based category	Number of CUI pairs (**X**,$\mathbf{Y}_{\mathbf{Tx}}$) with evidence-based category	Number of evidence-based information sources (Number of URIs) for CUI pairs (**X**,$\mathbf{Y}_{\mathbf{Tx}}$)	Number of CUI pairs (**X**,$\mathbf{Y}_{\mathbf{Tx}}$) with BMJ Best Practice as evidence source
Tx with therapeutic effect	190	73	117
Tx with uncertain therapeutic effect	38	22	11
Tx with unwanted or adverse effects	52	41	17
Potential Tx	5	5	0
Tx ingredient	22	21	6
General medical term	26	0	0
Correlation	94	108	19

Figure 2 in the main manuscript takes as source the data in Table 12. Although the 3CosAdd formula (a four-term type of embedding analogies not pairwise) brings concept pairs (**X**,$\mathbf{Y}_{\mathbf{Tx}}$) according to the seven evidence-based category utilised in this study (first column of Table 12), it is quite clear from Table 12 that the evidence-based category with a higher number of candidate concepts $\mathbf{Y}_{\mathbf{Tx}}$ is "Tx with therapeutic effect" with 190. For 117 of these 190 the evidence is taken from BMJ Best Practice that is acknowledged for its editorial quality and for evidence-based methodology [12].

The evidence-based category "general medical term" does not have evidence (zero information sources in Table 12) as it includes broad concepts $\mathbf{Y}_{\mathbf{Tx}}$ of little value for clinicians. From Table 12, it is noticeable that the evidence-based category with the highest number of evidence-based information sources is "correlation" with 108 of the total 238 evidence-based information sources utilised in this study.

Table 13 and 14 provide further details about the number of evidence-based information sources utilised in this study. The same evidence-based information source can provide evidence (quotes) for more than one concept pair (**X**,$\mathbf{Y_{Tx}}$). For example, ten topics from BMJ Best practice (i.e. ten documents each one with a unique URI) provide evidence for 117 UMLS CUI pairs (**X**,$\mathbf{Y_{Tx}}$) under "Tx with therapeutic effect".

Table 13 – Number of evidence-based information sources (Number of URIs) per disease target **x**

Disease Target **x**	Number of evidence-based information sources (Number of URIs)	Number of evidence-based information sources (Number of URIs)					
		Tx with therapeutic effect	Tx with uncertain therapeutic effect	Tx with unwanted or adverse effects	Potential Tx	Tx ingredient	Correlation
heart_failure	20	3	2	6	0	4	8
glaucoma	11	8	0	1	0	0	4
CKD	6	5	0	0	0	0	2
diabetes	5	4	0	1	0	3	0
asthma	32	10	5	5	1	3	11
epilepsy	56	7	3	17	2	5	25
arthritis	17	2	3	2	0	1	10
osteoarthritis	42	19	5	2	0	4	23
anaemia	18	8	1	1	0	0	9
hypertension	31	7	3	6	2	1	16
Total	238	73	22	41	5	21	108

Table 14 – Number of CUI pairs (**X**,$\mathbf{Y_{Tx}}$) with BMJ Best Practice as evidence source per disease target **x**. Each topic number has a unique URI, e.g. the URI http://bestpractice.bmj.com/topics/en-gb/61 corresponds to topic: 61

Disease Target **x**	BMJ Best Practice (topic: number)	Number of CUI pairs (**X**,$\mathbf{Y_{Tx}}$) with BMJ Best Practice as evidence source					
		Tx with therapeutic effect	Tx with uncertain therapeutic effect	Tx with unwanted or adverse effects	Potential Tx	Tx ingredient	Correlation
heart_failure	topic: 61	15	0	2	0	3	0
glaucoma	topic: 373	4	0	0	0	0	4
CKD	topic: 84	5	0	0	0	0	1
diabetes	topic: 24	7	0	0	0	1	0
asthma	topic: 44	15	1	1	0	0	0
epilepsy	topic: 543	14	0	6	0	0	0
arthritis	topic: 105	9	0	2	0	0	0
osteoarthritis	topic: 192	18	10	2	0	2	2
anaemia	topic: 93	10	0	0	0	0	10
hypertension	topic: 26	20	0	4	0	0	2
Total	10	117	11	17	0	6	19

2.3.3 Cohen's Kappa (including paradox resolution)

Multimedia Appendix 2 (worksheet Stage3) has the 408 concept pairs (**X**,$\mathbf{Y_{Tx}}$) with evidence and the two observers agreement or disagreement. Observer O1 disagrees with 25 concept pairs with evidence and observer O2 disagrees with 26. Cohen's Kappa=-0.023 is paradoxical [26,32,33]. To resolve the paradox, we calculate "p_{poS} and p_{neg} as two separate indexes of proportionate agreement in the observers' positive and negative decisions" [33]. Applying the formulas in [33], the positive agreement p_{poS} is 0.937 and negative agreement p_{neg} is 0.040, and the discrepancy is immediately evident (disparate p_{poS} and p_{neg} values).

Each observer was further asked to provide an alternative evidence-based category when in disagreement (column L and N in Multimedia Appendix 2 worksheet Stage3). We calculate Cohen's Kappa [19] between observers as a binary classification with 1 when (**X**,$\mathbf{Y_{Tx}}$) has $\mathbf{Y_{Tx}}$ belonging to the evidence-based category "Tx with therapeutic

effect" either agreed or assigned by the observer and 0 otherwise. The Cohen's Kappa is 0.921 interpreted as "almost perfect agreement" [26].

2.3.4 Illustrating a best clinical winner that is also a NER winner

Table 15 shows a best clinical winner with Max(n4) = 8, which is also a NER winner with Max(n3) = 8, for CBOW and the search query **-asthma +inhaled_corticosteroids +inhaled_corticosteroid** with disease target **x** = asthma.

The penultimate column (right hand-side) in Table 15 shows the category for concept $\mathbf{Y}_{\mathbf{Tx}}$, considering the six main categories introduced based on 38 UMLS Semantic Types (see Table 6 for details).

Table 15 – Illustrating a best clinical winner that is also a NER winner. The search query **-asthma +inhaled_corticosteroids +inhaled_corticosteroid** for CBOW

Rank	Candidate **y**	3CosAdd	UMLS CUI for concept $\mathbf{Y}_{\mathbf{Tx}}$ mapped to candidate $\mathbf{y}_{\mathbf{Tx}}$	Categories for concept $\mathbf{Y}_{\mathbf{Tx}}$ (UMLS CUI) based on its UMLS Semantic Types	Evidence-based categories for concept $\mathbf{Y}_{\mathbf{Tx}}$ correlated with concept **X**
1	formoterol	0.464184	C0060657	Substance	Tx with therapeutic effect
2	chronic_asthma	0.433854			
3	ICS	0.422363	C4020618	T061 or T121	Tx with therapeutic effect
4	fluticasone	0.410841	C0082607	Substance	Tx with therapeutic effect
5	tiotropium	0.410794	C0213771	Substance	Tx with therapeutic effect
6	salmeterol	0.408642	C0073992	Substance	Tx with therapeutic effect
7	beta2-agonist	0.407394	C2936789	T061 or T121	Tx with therapeutic effect|Tx with unwanted or adverse effects (i.e. non-therapeutic)
8	long-acting_beta-agonist	0.399669	C0001644	T061 or T121	Tx with therapeutic effect
9	long-acting_beta-agonists	0.398853	C0001644	T061 or T121	Tx with therapeutic effect
10	ipratropium	0.393822	C0027235	Substance	Tx with therapeutic effect
11	budesonide/formoterol	0.391721	C1276807	T061 or T121	Tx with therapeutic effect
12	long_acting	0.380315			

2.3.5 Source data for Figure 3

Table 16 shows the performance of the heuristic for 304 search queries considering: a) the values of n4 (the last three yellow columns in Multimedia Appendix 2 worksheet Q4); b) different thresholds for n4; and c) precision and recall as a metric.

Table 16 – Precision and Recall for the empirical heuristic developed with the 304 search queries from Multimedia Appendix 2 worksheet Q4

Threshold n4 >	TP	FP	FN	Precision %	Recall %
0	91	2	189	97.85	32.50
1	84	9	151	90.32	35.74
2	73	20	111	78.49	39.67
3	48	45	76	51.61	38.71
4	28	65	52	30.11	35.00
5	14	79	20	15.05	41.18
6	9	84	5	9.68	64.29
7	4	89	0	4.30	100.00

3. Cross-comparison with reusable datasets for evaluating relatedness

We investigate the UMLS CUI pairs among the reusable datasets for evaluating relatedness from [34,35] and the MRREL table [31] for UMLS 2019AA. We look for CUI pairs (**X**,**Y**) or (**Y**,**X**) where:

- UMLS Metathesaurus concept **X** has the UMLS Semantic Type "T047|Disease or Syndrome".
- UMLS Metathesaurus concept **Y** has the UMLS Semantic Type "T061|Therapeutic or Preventive Procedure" or "T121|Pharmacologic Substance".

The reusable datasets for evaluating relatedness from [34,35] and modified versions of them can be downloaded from [36] as CVS files. Table 17 shows the total number of CUI pairs and the number of CUI pairs with the UMLS Semantic Types –mentioned above.

Table 17 – Number of UMLS CUI pairs among the reusable datasets for evaluating relatedness from [36]

CVS file name	PubMed ID (PMID)	Total number of CUI pairs	Number of CUI pairs (**X**,**Y**) or (**Y**,**X**)
MayoSRS	16875881	101	6
MiniMayoSRS	16875881	29	0
UMNSRS_relatedness	21347043	587	65
UMNSRS_relatedness_mod458_word2vec	27531100	458	53

Relatedness is interpreted as an association existing between the CUI pairs. The 29 CUI pairs within the MiniMayoSRS.csv file (appears in the first column in Table 17) are the concept pairs from the MayoSRS.csv with the high interrater agreement for semantic relatedness.

The seven evidence-based categories introduced in this study provide a more fine-grained semantics for the relatedness (association) among the 408 concept pairs ($\mathbf{X}$,$\mathbf{Y}_{\mathbf{Tx}}$) investigated thoroughly, and enrich with evidence (quotes and references) from the biomedical literature. Table 18 and 19 show the 408 concept pairs ($\mathbf{X}$,$\mathbf{Y}_{\mathbf{Tx}}$) according to the evidence-based category assigned. For two external observers (O1 and O2) and applying the formulas in [33], the positive agreement p_{poS} is 0.937 (quite close to the highest value achievable 1.00) for the 408 concept pairs ($\mathbf{X}$,$\mathbf{Y}_{\mathbf{Tx}}$).

A comparison between the last columns of Table 17 and 18 indicates the number of CUI pairs for the evidence-based category "Tx with therapeutic effect" is 89 and above the highest number 65 of CUI pairs from [34,35].

Table 18 – Number of UMLS CUI pairs ($\mathbf{X}$,$\mathbf{Y}_{\mathbf{Tx}}$) according to the seven evidence-based categories introduced. The last column shows the number of CUI pairs ($\mathbf{X}$,$\mathbf{Y}_{\mathbf{Tx}}$) where $\mathbf{Y}_{\mathbf{Tx}}$ has the UMLS Semantic Type T061 or T121

Evidence-based category	Number of CUI pairs ($\mathbf{X}$,$\mathbf{Y}_{\mathbf{Tx}}$) with evidence	Number of CUI pairs ($\mathbf{X}$,$\mathbf{Y}_{\mathbf{Tx}}$) with evidence and concept $\mathbf{Y}_{\mathbf{Tx}}$ with Semantic Type T061 or T121
Tx with therapeutic effect	190	89
Tx with uncertain therapeutic effect	38	15
Tx with unwanted or adverse effects	52	21
Potential Tx	5	3
Tx ingredient	22	4
General medical term	26	21
Correlation	94	54

Finally, we investigated how many of the 408 concept pairs ($\mathbf{X}$,$\mathbf{Y}_{\mathbf{Tx}}$) investigated thoroughly, and enrich with evidence (quotes and references) from the biomedical literature, appear in the MRREL table [31] for UMLS 2019AA. Table 19 shows that only 45 of the 190 UMLS CUI pairs ($\mathbf{X}$,$\mathbf{Y}_{\mathbf{Tx}}$) with the evidence-based category "Tx with therapeutic effect" appear within MRREL table of UMLS 2019AA, which incorporates the MED-RT (Medication Reference Terminology) [37]. This fact seems to corroborate the difficulty for clinicians to obtain comprehensive information of the clinical worth of alternative choices for treating a given condition [38].

Table 19 – Number of UMLS CUI pairs ($\mathbf{X},\mathbf{Y}_{Tx}$) according to the seven evidence-based categories introduced. The last column shows the number of CUI pairs ($\mathbf{X},\mathbf{Y}_{Tx}$) or ($\mathbf{Y}_{Tx}$,$\mathbf{X}$) in MRREL table [31] of UMLS 2019AA

Evidence-based category	Number of CUI pairs ($\mathbf{X},\mathbf{Y}_{Tx}$) with evidence	Number of UMLS CUI pairs ($\mathbf{X}$, $\mathbf{Y}_{Tx}$) or ($\mathbf{Y}_{Tx}$, $\mathbf{X}$) in MRREL table of UMLS 2019AA
Tx with therapeutic effect	190	45
Tx with uncertain therapeutic effect	38	5
Tx with unwanted or adverse effects	52	9
Potential Tx	5	0
Tx ingredient	22	0
General medical term	26	0
Correlation	94	4

References

[1] Thomo A. Latent Semantic Analysis (Tutorial). https://www.engr.uvic.ca/~seng474/svd.pdf

[2] Levy O, Goldberg Y. Linguistic regularities in sparse and explicit word representations. Proceedings of the 18th conference on computational natural language learning. 2014;171-180. doi: 10.3115/v1/W14-1618

[3] Mikolov T, Chen K, Corrado G, Dean J. Efficient estimation of word representations in vector space. Proceedings of 1st International Conference on Learning Representations (ICLR 2013). 2013

[4] word2vec. https://code.google.com/archive/p/word2vec/source/default/source

[5] The 5 lines of text as word2vec input. http://semdeep.cs.man.ac.uk/JMIR_embedding_analogies_2019/Romeo_inputDocs.txt

[6] Cruse A. Meaning in Language: An Introduction to Semantics and Pragmatics. Oxford University Press UK. 2011. ISBN: 9780199559466

[7] Nerlich B, Clarke DD. Semantic fields and frames: Historical explorations of the interface between language, action, and cognition. Journal of pragmatics. 2000;32(2):125-50. doi: 10.1016/S0378-2166(99)00042-9

[8] Additional file 1 and 3: worksheet "PMSB". https://jbiomedsem.biomedcentral.com/articles/10.1186/s13326-019-0212-6#MOESM1

[9] Bodenreider O. The Unified Medical Language System (UMLS): integrating biomedical terminology. Nucleic Acids Res. 2004; 32(Database issue): D267–D270. PMID: 14681409

[10] BMJ Best Practice. https://bestpractice.bmj.com/

[11] PubMed/MEDLINE. https://www.ncbi.nlm.nih.gov/pubmed/

[12] Kwag KH, González-Lorenzo M, Banzi R, Bonovas S, Moja L. Providing Doctors With High-Quality Information: An Updated Evaluation of Web-Based Point-of-Care Information Summaries. J Med Internet Res. 2016;18(1):e15. PMID: 26786976

[13] Free access of NHS professionals to BMJ Best Practice. https://bestpractice.bmj.com/info/bmagp/

[14] Donnelly K. SNOMED-CT: The advanced terminology and coding system for eHealth. Stud Health Technol Inform. 2006;121:279-90. PMID: 17095826

[15] Nadkarni PM, Ohno-Machado L, Chapman WW. Natural language processing: an introduction. J Am Med Inform Assoc. 2011;18(5):544-51. PMID: 21846786

[16] Yamamoto Y, Yamaguchi A, Bono H, Takagi T. Allie: a database and a search service of abbreviations and long forms. Database (Oxford). 2011 . PMID: 21498548

[17] Hart T, Tsaousides T, Zanca JM, Whyte J, Packel A, Ferraro M, Dijkers MP. Toward a theory-driven classification of rehabilitation treatments. Arch Phys Med Rehabil. 2014;95(1):S33-44.e2. PMID: 24370323

[18] Artstein R ,Poesio M. Inter-coder agreement for computational linguistics. Computational Linguistics. 2008; 34(4):555-596.

[19] Cohen J. A coefficient of agreement for nominal scales. Educational and Psychological Measurement. 1960; 20(1):37–46.

[20] Krippendorff K. Content Analysis: An Introduction to Its Methodology. 1980. ISBN: 9781506395661

[21] Rebholz-Schuhmann D, Oellrich A, Hoehndorf R. Text-mining solutions for biomedical research: enabling integrative biology. Nat Rev Genet. 2012;13(12):829-39. PMID: 23150036

[22] Aronson AR, Lang FM. An overview of MetaMap: historical perspective and recent advances. J Am Med Inform Assoc. 2010;17(3):229-36. PMID: 20442139

[23] NER guidelines for MetaMap's output. https://static-content.springer.com/esm/art%3A10.1186%2Fs13326-019-0212-6/MediaObjects/13326_2019_212_MOESM2_ESM.pdf

[24] Manning CD, Schütze H. Foundations of statistical natural language processing. MIT press. 1999. ISBN: 9780262133609

[25] Pratt W, Yetisgen-Yildiz M. A study of biomedical concept identification: MetaMap vs. people. AMIA Annu Symp Proc. 2003;529-33. PMID: 14728229

[26] Viera AJ, Garrett JM. Understanding interobserver agreement: the kappa statistic. Fam Med. 2005;37(5):360-3. PMID: 15883903

[27] UMLS Semantic Types. https://metamap.nlm.nih.gov/Docs/SemanticTypes_2013AA.txt

[28] Cabrera-Perez J, Condotta SA, Badovinac VP, Griffith TS. Impact of sepsis on CD4 T cell immunity. J Leukoc Biol. 2014 Nov;96(5):767-77. PMID: 24791959

[29] The semantic science integrated ontology (SIO). http://semanticscience.org/ontology/sio.owl

[30] Ries M, Gal A. Genotype–phenotype correlation in Fabry disease. In: Mehta A, Beck M, Sunder-Plassmann G, editors. Fabry Disease: Perspectives from 5 Years of FOS. Oxford: Oxford PharmaGenesis; 2006. Chapter 34. PMID: 21290681

[31] MRREL table. https://www.ncbi.nlm.nih.gov/books/NBK9685/table/ch03.T.related_concepts_file_mrrel_rrf/

[32] Feinstein AR, Cicchetti DV. High agreement but low kappa: I. The problems of two paradoxes. J Clin Epidemiol. 1990;43(6):543-9. PMID: 2348207

[33] Cicchetti DV, Feinstein AR. High agreement but low kappa: II. Resolving the paradoxes. J Clin Epidemiol. 1990;43(6):551-8. PMID: 2189948

[34] Pedersen T, Pakhomov SV, Patwardhan S, Chute CG. Measures of semantic similarity and relatedness in the biomedical domain. J Biomed Inform. 2007;40(3):288-99. PMID: 16875881

[35] Pakhomov S, McInnes B, Adam T, Liu Y, Pedersen T, Melton GB. Semantic Similarity and Relatedness between Clinical Terms: An Experimental Study. Proc. AMIA Annu Symp 2010:572-6. PMID: 21347043

[36] Reusable datasets for evaluating semantic similarity and relatedness. http://rxinformatics.umn.edu/SemanticRelatednessResources.html

[37] MED-RT (Medication Reference Terminology). https://www.nlm.nih.gov/research/umls/sourcereleasedocs/current/MED-RT/

[38] Avorn J. The Psychology of Clinical Decision Making - Implications for Medication Use. N Engl J Med. 2018;378(8):689-691. PMID: 29466158
